# IL-33/ST2 pathway drives regulatory T cell dependent suppression of liver damage upon cytomegalovirus infection

**DOI:** 10.1371/journal.ppat.1006345

**Published:** 2017-04-27

**Authors:** Branka Popovic, Mijo Golemac, Jürgen Podlech, Jelena Zeleznjak, Lidija Bilic-Zulle, Miodrag L. Lukic, Luka Cicin-Sain, Matthias J. Reddehase, Tim Sparwasser, Astrid Krmpotic, Stipan Jonjic

**Affiliations:** 1 Department of Histology and Embryology, Faculty of Medicine, University of Rijeka, Rijeka, Croatia; 2 Institute for Virology and Research Center for Immunotherapy, University Medical Center of the Johannes Gutenberg-University, Mainz, Germany; 3 Clinical Institute of Laboratory Diagnostics, Clinical Hospital Center, Rijeka, Croatia; 4 Department of Microbiology and Immunology, Centre for Molecular Medicine and Stem Cell Research, Faculty of Medicine, University of Kragujevac, Kragujevac, Serbia; 5 Department of Vaccinology and Applied Microbiology, Helmholtz Centre for Infection Research (HZI), Braunschweig, Germany; 6 German Center for Infection Research (DZIF), Hannover-Braunschweig site, Braunschweig, Germany; 7 Institute of Infection Immunology, TWINCORE, Hannover, Germany; La Jolla Institute for Allergy and Immunology, UNITED STATES

## Abstract

Regulatory T (Treg) cells dampen an exaggerated immune response to viral infections in order to avoid immunopathology. Cytomegaloviruses (CMVs) are herpesviruses usually causing asymptomatic infection in immunocompetent hosts and induce strong cellular immunity which provides protection against CMV disease. It remains unclear how these persistent viruses manage to avoid induction of immunopathology not only during the acute infection but also during life-long persistence and virus reactivation. This may be due to numerous viral immunoevasion strategies used to specifically modulate immune responses but also induction of Treg cells by CMV infection. Here we demonstrate that liver Treg cells are strongly induced in mice infected with murine CMV (MCMV). The depletion of Treg cells results in severe hepatitis and liver damage without alterations in the virus load. Moreover, liver Treg cells show a high expression of ST2, a cellular receptor for tissue alarmin IL-33, which is strongly upregulated in the liver of infected mice. We demonstrated that IL-33 signaling is crucial for Treg cell accumulation after MCMV infection and ST2-deficient mice show a more pronounced liver pathology and higher mortality compared to infected control mice. These results illustrate the importance of IL-33 in the suppressive function of liver Treg cells during CMV infection.

## Author summary

Treg cells are crucial for immune homeostasis and for dampening immune response to several diseased conditions, including viral infections. Murine cytomegalovirus (MCMV) is a herpesvirus with pathogenic potential, so that early immune mechanisms are essential in controlling virus and protecting from virus-induced pathology. Studies on Foxp3^+^ Treg cells have revealed their inhibitory role on the early T cell response to MCMV infection and have suggested Treg cells as a target of MCMV’s immunoevasion mechanisms. Here we demonstrate that the number and activation status of liver Treg cells is strongly induced upon MCMV infection in order to protect the host from severe liver damage. They constitutively express high amounts of IL-33 receptor ST2 and their accumulation depends on IL-33, which is released as a tissue alarmin after the cell damage. For the first time, we show an immunoregulatory role of IL-33-dependent Treg cells in the liver of MCMV infected mice and their suppression of MCMV-induced immunopathology.

## Introduction

Regulatory CD4^+^Foxp3^+^ T (Treg) cells play an essential role in maintaining immune homeostasis and suppressing an overwhelming immune response in several diseased conditions including viral infections and cancer. The transcription factor Foxp3 is essential for Treg cell differentiation and function, thus a mutation in the *Foxp3* gene results in an immune-mediated disorder affecting multiple organs in both mice and humans [1]. Beside the naturally occurring Treg cells (nTreg) which mature in the thymus, a variety of induced Treg cells (iTreg) arise from naive CD4^+^Foxp3^−^ T cells in the periphery, under influence of tissue microenvironment and cytokines [2]. Treg cells employ various immunoregulatory mechanisms including the inhibition of antigen presenting cell function, a direct killing of effector cells, the consumption of IL-2 and the production of immunosuppressive cytokines such as IL-10, TGFβ and IL-35 or amphiregulin [3–5]. However, the phenotype of Treg cells and their suppressive mechanisms differ depending on particular tissue and disease settings [3]. For example, certain subsets of Treg cells, specifically those in adipose tissue and intestines, express high amounts of the IL-33 receptor ST2, and require IL-33 for their maintenance and suppressive function [6]. Tissue alarmin IL-33 has been associated with the differentiation and function of various lymphocytes including Treg cells. In addition to T helper 2 (Th2) cells, Treg cells constitutively express high amounts of ST2, unlike other CD4^+^ and CD8^+^ T cell subsets [7].

Several studies have described the involvement of Treg cells in the immune response to viral infections [8]. For instance, Treg cells can modulate early T-cell trafficking to infected nonlymphoid sites and facilitate protective responses against herpes simplex virus (HSV), lymphocytic choriomeningitis virus (LCMV) and respiratory syncytial virus (RSV) infection [9, 10]. On the other hand, Treg cells can reduce the effector T-cell response and inhibit anti-viral cytokine production [8]. Although the suppression of an excessive immune response is beneficial for the host since it limits immunopathology, the suppression of an early response could adversely affect virus control. Thus, some viruses are able to expand activity and number of Treg cells as a mechanism to escape from an effective immune response [11–13]. This has been suggested also for cytomegalovirus (CMV) which is well known for developing different immune evasion strategies aimed at avoiding immune cell recognition [14, 15]. The support for this idea came from several previous studies, which demonstrated that murine cytomegalovirus (MCMV) infection induces both nTreg and iTreg cells [16–18]. The depletion of Treg cells leads to an increased T cell response, major players in controlling an early MCMV infection [16, 17]. In addition, Treg depletion results in reduced viral titers in salivary glands of BALB/c mice [16, 17] highlighting these cells as a target of immune evasion. However, recent studies link human Treg expansion to a decreased vascular pathology in CMV infected elderly individuals [19] and the depletion of mouse Treg cells in MCMV infected brain augmented chronic gliosis [18]. Thus, it remains unclear whether Foxp3^+^ Treg cells function in a positive way to limit an exaggerated immune activation and consequent CMV-induced immunopathology.

Here we aimed to determine if the host benefits from an early induction of Foxp3^+^ Treg cells upon MCMV infection. Particularly, we were interested to determine whether these cells can counteract MCMV-induced liver damage. Our data demonstrate that MCMV infection induces both splenic and liver Treg cells. However, the activation and proliferation of Treg cells is much more pronounced in the liver compared to the spleen. In addition, Treg deficiency results in severe liver pathology in infected mice. Similar results were obtained in mice lacking the IL-33 receptor, with an impaired accumulation of Treg cells in the liver following infection. Together, our data unveil the importance of IL-33-dependent Treg cells in preventing MCMV-induced liver damage.

## Results

### MCMV infection leads to the accumulation of activated Treg cells in the liver

**Fig 1:**
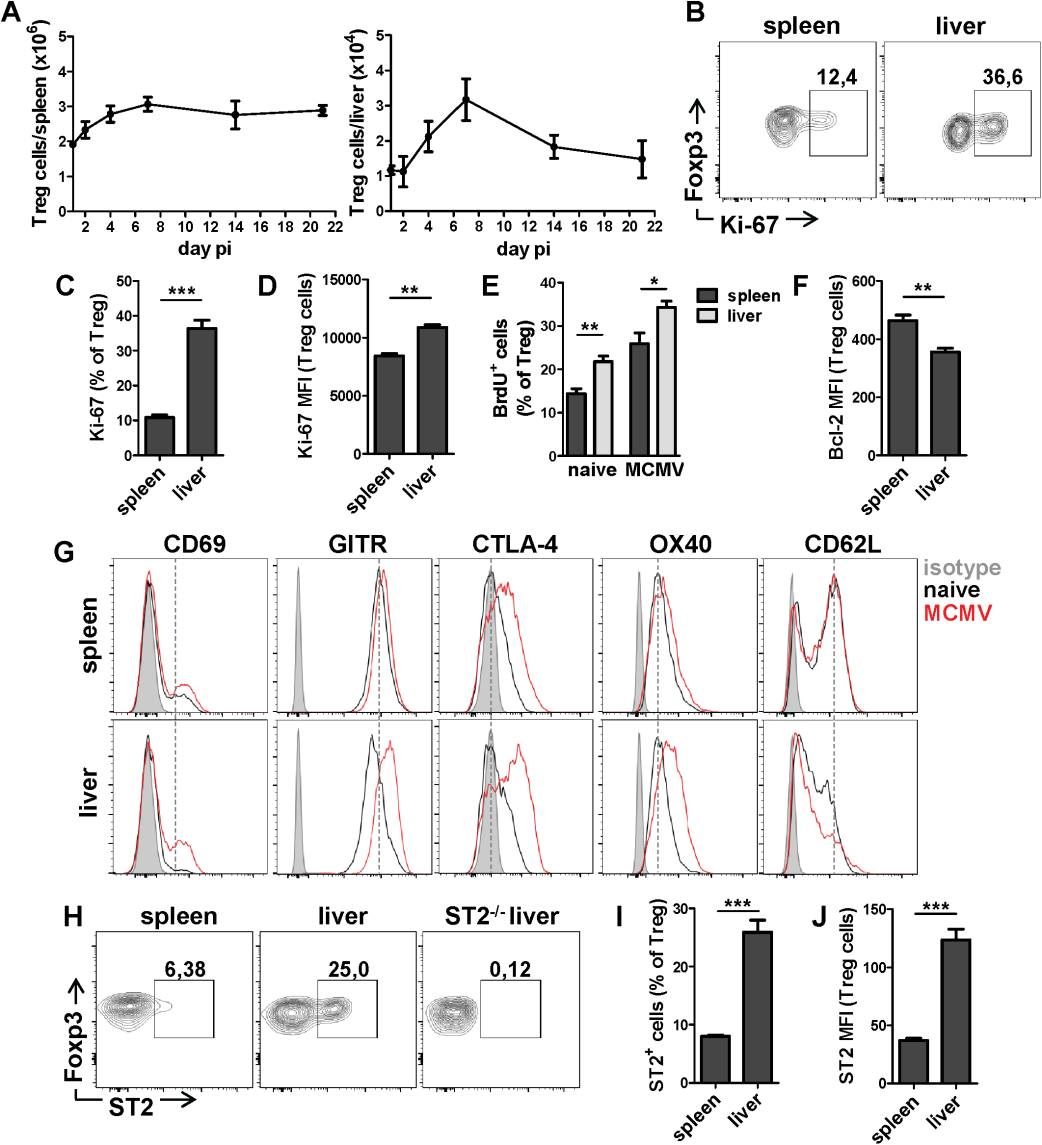
Treg cells show an activated phenotype after MCMV infection. BALB/c mice were i.v. injected with 2x10^5^ PFU of WT MCMV (clone MW97.01) or left uninfected. (**A**) Absolute number of Treg cells in spleen and liver is shown. (**B**) Representative FACS plots and (**C**) graphs showing percentages and (**D**) median fluorescence intensity (MFI) of Ki-67 expression by naive Treg cells. (**F**) Bcl-2 expression by naive Treg cells. (**E**) Mice were treated with BrdU in drinking water for 6 days starting at the day of infection. Percentage of BrdU positive Treg cells on day 7 was determined. (**G**) Histograms show a representative expression of different markers by Treg cells from uninfected and 7 days infected mice. (**H**) Representative FACS plots and (**I**) graphs showing percentages and (**J**) median fluorescence intensity (MFI) of ST2 expression by Treg cells isolated from the spleen and liver of naive BALB/c and ST2^-/-^ mice. Data are shown as mean ± SEM of n = 3–5 mice from one representative experiment out of three. *p<0.05; ** p<0.01; ***p<0.001 from two tailed, unpaired Student’s t-test.

To assess the impact of acute MCMV infection on the Treg cell response we have characterized in detail the kinetics and phenotype of Treg cells in the spleen and liver, two major target organs for MCMV replication. For this, we have infected naive BALB/c mice intravenously (i.v.) with WT MCMV and analyzed Treg cell responses in both spleen and liver at day 1.5, 4, 7, 14 and 21 post infection (p.i.). We have observed a significant increase in the absolute numbers of these cells in the acute phase of infection which peaked at day 7 p.i. in both organs (Fig 1A). A similar trend has been observed when percentages of Treg cells were assessed, particularly in the liver (S1A Fig). The expansion was followed by a contraction phase in the liver similar to MCMV specific non-inflationary CD8^+^ and CD4^+^ T cell responses [20], whereas splenic Treg cells were maintained at high numbers even 3 weeks after infection. To determine the proliferative capacity of naive Treg cells from either the spleen or the liver, we have measured the expression of proliferation-associated nuclear antigen Ki-67. A significantly higher percentage of liver Treg cells expressed Ki-67 with a higher median fluorescence intensity (MFI) compared to splenic Treg cells (Fig 1B–1D). In line with a higher Ki-67 expression, liver Treg cells exhibited a significantly higher incorporation of BrdU than splenic Treg cells in naive but also MCMV infected mice confirming their enhanced proliferation (Fig 1E). In contrast, splenic Treg cells expressed significantly higher levels of the anti-apoptotic protein Bcl-2 than did liver Treg cells (Fig 1F). Notably, an inverse correlation between expression of Bcl-2 and Ki-67 by liver and splenic Treg cells was observed. The large majority of Ki-67 positive Treg cells were also positive for Helios, i.e. belong to nTreg subset (S1B Fig). We have further characterized splenic and liver Treg cells in MCMV infected mice and assessed the expression of several cell surface molecules. Based on an elevated expression of activation markers on Treg cells, such as CD69, GITR, CTLA-4 and OX-40, Treg cells from 7-days infected mice showed an activated phenotype (Fig 1G). Interestingly, the difference in activation between naive and MCMV-induced Treg cells was even more pronounced in the liver than in the spleen of the same mice, with a corresponding very low expression of the homing receptor L-selectin (CD62L). The secretion of immunosuppressive cytokines and cytolytic granzyme B is a well described mechanism by which Treg cells exert their suppressive activity [21]. To characterize their production by Treg cells in MCMV infected mice, we have determined the frequency of Treg cells expressing IL-10, the latency associated protein (LAP), a part of TGFβ precursor, its cell surface receptor GARP, and granzyme B in spleen and liver. The expression of IL-10, LAP/TGFβ, GARP and cytolytic granyzme B by Treg cells was induced after MCMV infection. While IL-10 production was detected only in Foxp3 negative CD4^+^ cells, LAP/TGFβ and GARP were predominantly expressed by CD4^+^Foxp3^+^ cells (S1C Fig). Unlike IL-10 and TGFβ, granzyme B was expressed in a proportion of both subsets. Altogether, MCMV infection results in expansion of Treg cells with a more activated phenotype in the liver compared to the spleen.

### Enrichment of ST2^+^ Treg cells in liver

Previous studies have shown the importance of IL-33 in Treg cell induction and tissue-dependent expression of IL-33 receptor ST2 by Treg cells [6]. Using flow-cytometric analysis of splenic and liver Treg cells from BALB/c and ST2^-/-^ mice we have shown an enrichment of ST2-expressing Treg cells in the liver compared to the spleen of BALB/c mice, measured as either the fraction of ST2^+^ cells or ST2 MFI (Fig 1H–1J). Next, we have determined the kinetics of ST2^+^ Treg cells after MCMV infection and showed an increase of ST2^+^ Treg cells upon infection in both organs (S1D Fig). To determine ST2-related effects on Treg cell proliferation, we have measured Ki-67 expression and the incorporation of BrdU by ST2^+^ and ST2^-^ Treg cells. The proliferation of ST2^+^ Treg cells was clearly upregulated compared to ST2^-^ Treg cells, with a reduced Bcl-2 expression (S2A and S2B Fig). The majority of ST2^+^ Treg cells were thymus derived and expressed Helios and Neuropilin-1 (S2C Fig). Next, we further compared the phenotypic characteristics of ST2^+^ and ST2^-^ Treg cells (S2D Fig) from the liver of naive BALB/c mice and this revealed that ST2^+^ Treg cells, while sharing the expression of classical Treg markers, are distinct from their ST2^-^ counterparts. Namely, ST2^+^ Treg cells were mostly CD62L^lo^ and exhibited an elevated expression of CD103, KLRG1, CTLA-4, GITR and PD-1 compared to their ST2 negative counterparts, resembling a previously described phenotype of splenic ST2^+^ Treg cells [22, 23]. A differential expression of these markers on ST2^+^ Treg cells was also observed (maintained) during MCMV infection (S2E Fig). Our data point to a constitutively higher frequency of Treg cells expressing ST2 in the liver with a distinct phenotype from ST2 negative Treg cells.

### Treg cells prevent severe liver damage upon MCMV infection

**Fig 2:**
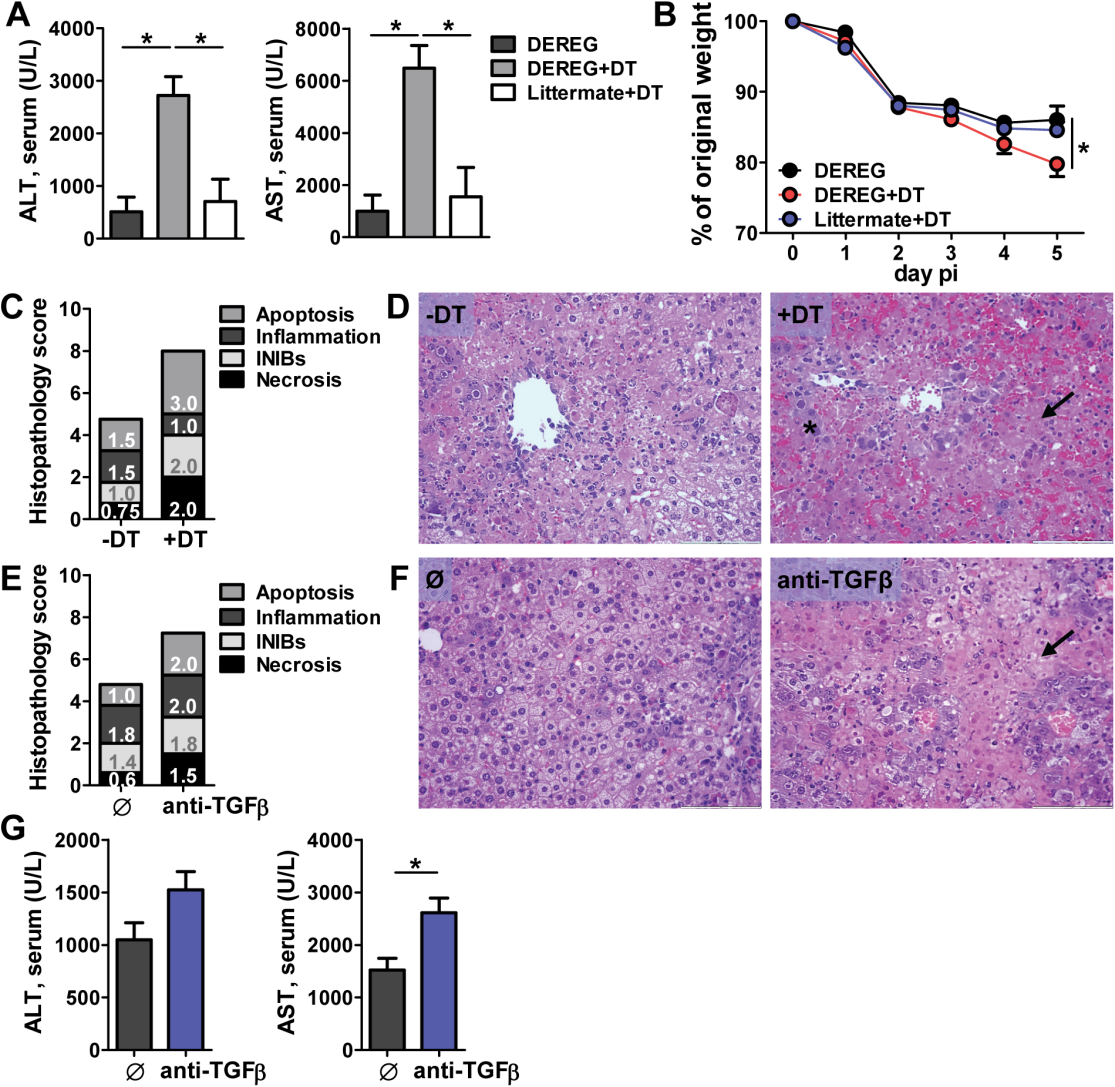
Depletion of Treg cells results in severe liver damage upon MCMV infection. (**A**-**D**) BALB/c DEREG mice were i.v. injected with 10^6^ WT MCMV (pSM3fr-MCK-2fl clone 3.3) and treated i.p. with DT on day 0 and 1 or left untreated. Mice were analyzed on day 5 p.i. (**A**) AST and ALT levels were determined in the serum (**B**) Changes in the body weight were monitored daily and plotted as percent of weight at the date of infection. (**C**) Scores of cumulative liver pathology for apoptosis, intranuclear inclusion bodies (INIBs), inflammation, and necrosis. Bars correspond to the mean score for each parameter. The height of each bar represents the mean of the total histological score (out of 12). (**D**) Representative H&E staining of paraffin embedded liver sections. Arrows highlight necrotic areas, and asterisks indicate hepatocytes with large nuclear inclusions. (**E**-**G**) BALB/c mice were i.p. injected with anti-TGFβ antibody 3 hours prior to i.v. infection with 10^6^ WT MCMV (pSM3fr-MCK-2fl clone 3.3) and analyzed on day 5 p.i. (**E**) Histology score and (**F**) representative H&E staining of liver sections. (**G**) AST and ALT levels in the serum. Data are shown as mean ± SEM of n = 3–5 mice from one representative experiment out of three. *p<0.05 from one-way ANOVA with Bonferroni correction and two tailed, unpaired Student’s t-test.

Treg cell activation and expansion has been proposed as a viral immune evasion strategy to avoid immune cell control [13]. However, Treg cells are also crucial for the maintenance of peripheral tolerance and prevention of autoimmunity; therefore, their role during viral infection is also beneficial for the host. To investigate the possible beneficial role of Treg cells during the acute phase of CMV infection, we have assessed the functional and histological parameters of liver damage following infection. Liver is one of the organs strongly affected with CMV infection where human cytomegalovirus (HCMV) can cause clinically relevant damage, leading to chronic hepatitis, cirrhosis and possible death in neonates or immunocompromised patients [24, 25]. MCMV causes liver damage in mice which is comparable to the damage in HCMV infected individuals including elevated liver enzymes, hepatitis and hepatocellular necrosis [26]. To assess the role of Treg cells in the liver, we have carried out the depletion of Treg cells in BALB/c mice by administration of anti-CD25 antibody 2 days prior to infection. This administration of anti-CD25 resulted in a significant elevation of aspartate aminotransferase (AST) and a modest increase in alanine aminotransferase (ALT) levels in the serum of treated mice compared to untreated, with no difference in virus titers (S3A and S3B Fig). However, the effects of anti-CD25 treatment can be misleading because despite the depletion of CD25^high^ cells a significant number of Foxp3^+^ cells remains [27]. In addition, activated effector T cells may transiently express CD25 and are thus potential targets for anti-CD25 antibodies [28]. Therefore, we have used BALB/c DEREG mice [29], which express the diphtheria toxin receptor under control of the Foxp3 locus, allowing for a selective depletion of Foxp3^+^ Treg cells by Diphtheria toxin (DT) administration. Mice were infected with MCMV and treated with DT on the day of infection and 1 day later, or left untreated, and analyzed 5 days p.i. Littermate controls treated with DT were used to exclude possible toxic and unspecific DT effects. Analysis of liver enzymes revealed significantly higher levels of AST and ALT in the serum of Treg-depleted mice compared to untreated mice, indicating a more severe liver damage in the absence of Treg cells in MCMV infected mice (Fig 2A). The uninfected DEREG mice did not develop any signs of liver pathology resulting from Treg ablation (S3C Fig). In addition, changes in body weight were monitored daily in DT-treated and control mice. Following MCMV infection, all groups of mice displayed a marked reduction in body weight, with highest loss observed in mice depleted of Treg cells (Fig 2B). Furthermore, histopathological analysis revealed a markedly increased severity and extent of overall tissue damage in the absence of Treg cells characterized by confluent areas of hepatocellular necrosis with mononuclear infiltrates and extravasation of erythrocytes (Fig 2C and 2D). To test whether this Treg effect is mediated by TGFβ secretion, we have injected BALB/c mice with TGFβ neutralizing antibodies prior to infection. Neutralization of TGFβ resulted in a 1.5-2-fold increase in liver enzymes at day 5 p.i. (Fig 2G) and histological analysis revealed a strongly induced liver pathology in treated mice (Fig 2E and 2F). Taken together, these data demonstrate that Treg cells and TGFβ can inhibit the development of severe liver damage during the acute phase of MCMV infection.

**Fig 3:**
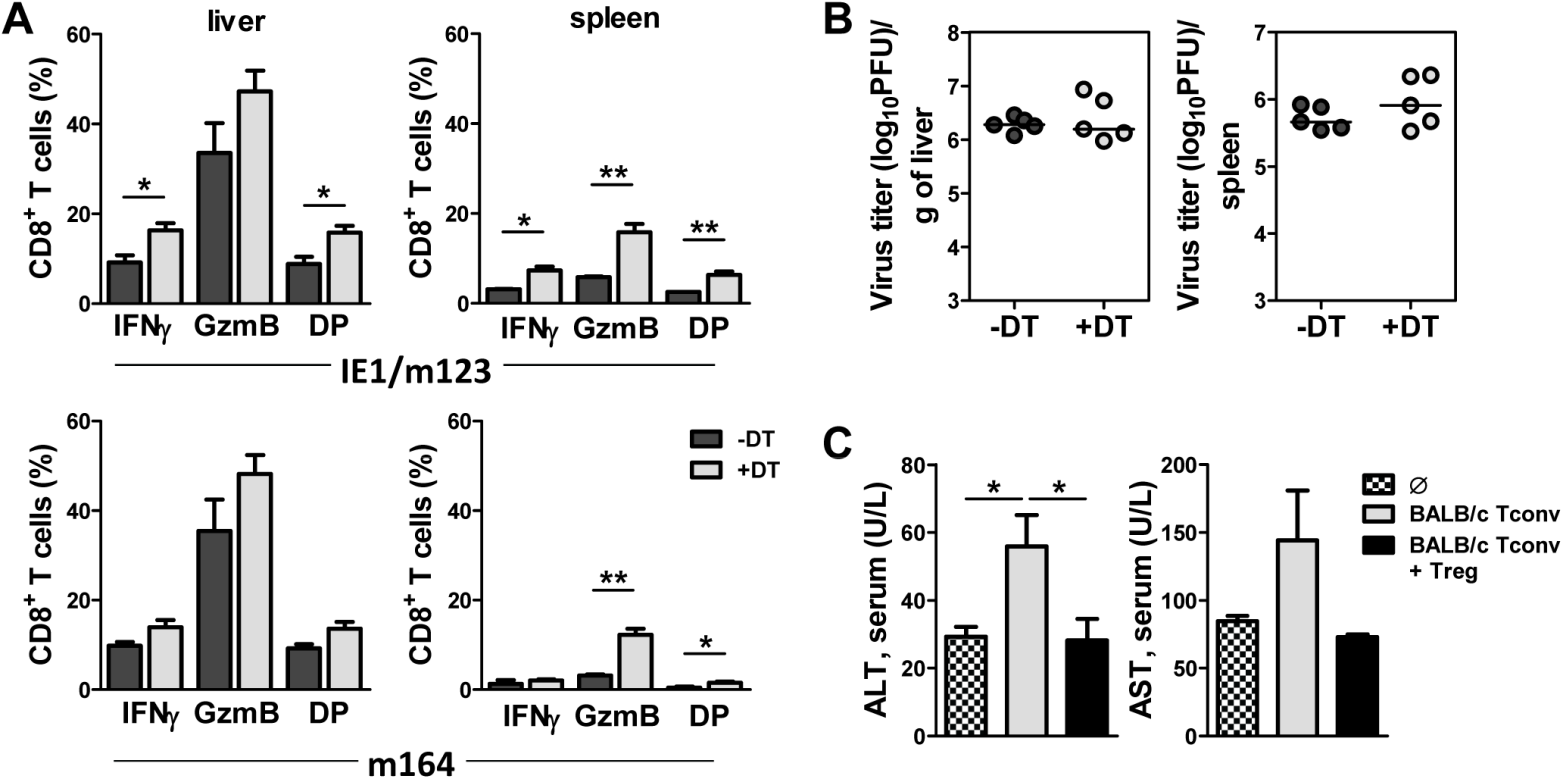
Depletion of Treg cells results in enhanced virus-specific CD8^+^ T cell response. BALB/c DEREG mice were i.v. injected with 10^6^ WT MCMV (pSM3fr-MCK-2fl clone 3.3) and treated i.p. with DT on day 0 and 1 or left untreated. Mice were analyzed on day 5 p.i. (**A**) Lymphocytes from spleen and liver were re-stimulated with IE1 or m164 peptides and frequency of CD8^+^ T cells expressing IFNγ, granzyme B, or both (double positive, DP), was determined. Data are shown as mean ± SEM of n = 3 mice from one representative experiment. *p<0.05; ** p < 0.01 from two tailed, unpaired Student’s t-test. (**B**) Viral titers in the spleen and liver were determined by the plaque assay. A circle depicts the titer for each individual mouse; a small horizontal line indicates the median. n = 5 (**C**) BALB/c SCID mice were i.v. injected with 10^6^ WT MCMV (pSM3fr-MCK-2fl clone 3.3) and at the same day of infection received 1x10^7^ splenocytes from naive BALB/c mice (anti-CD25 treated) alone or together with 1x10^6^ Treg cells. AST and ALT levels were determined in the serum on day 5 p.i. Data are shown as mean ± SEM of n = 5 mice from one representative experiment. *p<0.05 from one-way ANOVA with Bonferroni correction.

To assess whether TGFβ and Treg cells have overlapping influence on virus-induced liver pathology we have treated DEREG mice with TGFβ neutralizing antibodies prior to infection and DT injections. As expected, liver enzyme levels and weight loss on day 4 p.i. were similar in both groups of mice, indicating a Treg/TGFβ protecting axis during MCMV infection (S4A and S4B Fig). In the same experiment, we have treated mice with CD8 and CD4 depleting antibodies to show their putative role in liver damage. Mice depleted of either CD4^+^ or CD8^+^ T cells had similar levels of liver enzymes and weight loss as Treg-sufficient mice, although this was slightly more pronounced in animals depleted of CD8^+^ T cells. We have also measured the viral titers and CD8^+^ T cell response after re-stimulation with immunodominant viral peptides IE1 (immediate-early 1) and m164 [30, 31] in the liver and spleen of Treg depleted and nondepleted DEREG mice. Depletion of Treg cells resulted in a minor or no difference in viral loads (Fig 3B) but higher frequencies of IFNγ and granzyme B producing CD8^+^ T cells on day 5 p.i. in both organs (Fig 3A). Thus, Treg cells inhibit the MCMV-specific CD8^+^ T cell response in both the liver and spleen. To further determine the contribution of conventional T and Treg cells to liver damage, we have isolated splenocytes from naive BALB/c mice, pretreated with anti-CD25 antibody, and transferred them alone or together with purified Treg cells, to infected SCID mice. Mice that received only conventional T cells had 2-fold higher ALT and AST serum levels than untreated SCID mice. However, co-transfer of Treg cells rescued liver from MCMV induced damage to the same levels as in untreated SCID mice (Fig 3C). We have also adoptively transferred BALB/c CD8^+^ T cells alone or together with Treg cells to infected SCID mice. Transfer of only CD8^+^ T cells has induced a significant release of ALT, which was not the case in mice which have received Treg cells in combination with CD8^+^ T cells (S4C Fig). Altogether, these data demonstrate a protective role of Treg cells against T cell mediated MCMV-induced liver pathology.

### Increased IL-33 production in the liver of MCMV infected mice

**Fig 4:**
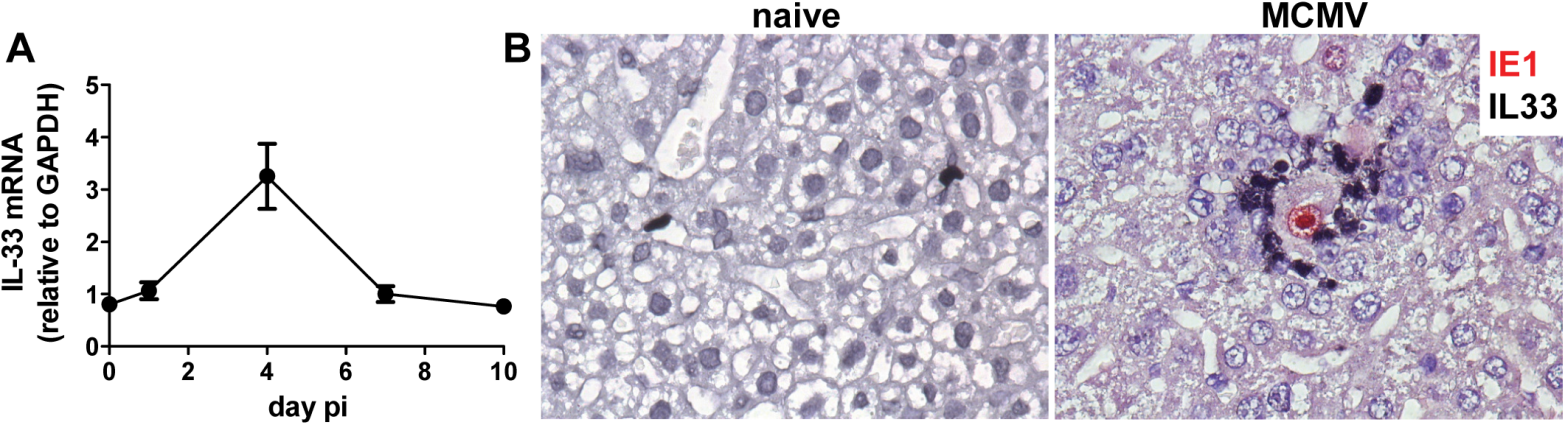
IL-33 expression is increased during MCMV infection. BALB/c mice were injected i.v. with 2x10^5^ PFU of WT MCMV (MW97.01) and liver tissue was harvested on indicated time points. (**A**) Kinetic analysis of IL-33 mRNA expression, relative to GAPDH mRNA, performed by real-time PCR. (**B**) Representative IL-33 (black) and MCMV IE1 (red) co-staining of paraffin embedded liver sections on day 5 p.i.

**Fig 5:**
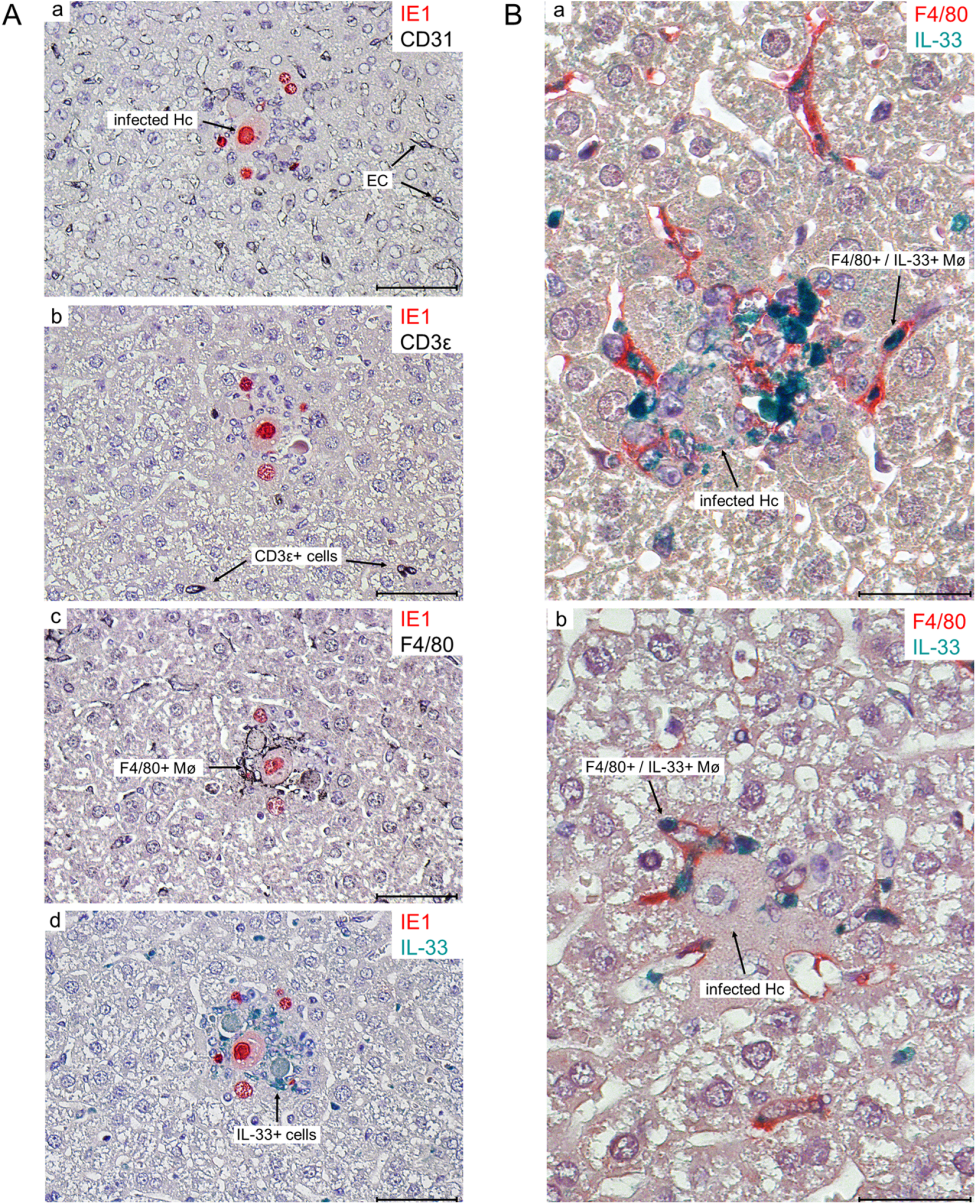
Identification of the cell type that expresses IL-33 in inflammatory foci in infected liver tissue as F4/80^+^ macrophages. BALB/c mice were injected i.v. with 5x10^5^ PFU of WT MCMV (MW97.01) and liver tissue was harvested on day 5 p.i. (**A**) Consecutive serial 1-μm sections of liver tissue focusing on an infected hepatocyte (Hc) that is delimited from uninfected tissue by a sheath made up by a mononuclear cell infiltrate. The expression of the indicated marker molecules was tested in a two-color IHC (2C-IHC) staining. (**a**-**d**) Identification of the infected Hc by red staining of the intranuclear viral IE1 protein. (**a**) Focus-forming mononuclear cells are not CD31^**+**^ black-stained endothelial cells (EC). (**b**) Focus-forming mononuclear cells are not CD3ε^**+**^ black-stained cells, thus excluding α/ß and γ/δ T cells as well as NKT cells. (**c**) Identification of focus-forming mononuclear cells as black-stained F4/80^**+**^ macrophages (Mø). (**d**) IL33-expressing cells stained in turquoise-green color colocalize with focus-forming F4/80 macrophages in the neighboring section of image **c**. Counterstaining with hematoxylin. Arrows point to the indicated cell types exemplarily. The bar markers represent 50 μm throughout. (**B**) 2C-IHC verifying colocalization of F4/80 and IL33 on the cellular level. (**a**) Higher resolution image of an advanced, aged focus consisting of a cluster of dual-stained F4/80^+^ (red) IL33^+^ (turquoise-green) macrophages (Mø) surrounding an infected hepatocyte (Hc) that is identified by an intranuclear inclusion body. Note that dually-expressing macrophages localize also to liver tissue outside of a focus. (**b**) A young focus in which dual-stained F4/80^**+**^ (red) IL33^+^ (turquoise-green) macrophages cling to an infected hepatocyte (Hc) that shows the pathocytomorphology of an owl’s eye cell with an intranuclear inclusion body that indicates the late phase (L phase) in the viral gene expression program. Counterstaining with hematoxylin. Arrows point to sites of interest. The bar markers represent 50 μm.

It has been well established that some viruses alter the expression of IL-33 during infection [32, 33]. Our goal was to assess the impact of MCMV infection on IL-33 expression and the consequent regulation of Treg cells. First, we have measured the expression of IL-33 by three different cell lines: B12 (SV40-transformed BALB/c fibroblasts), RAW 264.7 (A-MuLV-transformed BALB/c macrophages) and BALB/c LSEC (SV40-transformed liver sinusoidal endothelial cells). Cells were infected with virus lacking the *m138* gene in order to avoid unspecific interactions of the viral Fc receptor (encoded by *m138* gene) with the Fc portion of immunoglobulins. The expression of IL-33 was detected under normal conditions in all of the tested cell lines and increased 24 h post MCMV infection with the highest difference between uninfected and infected cells observed in LSECs (S5 Fig). In order to dissect the *in vivo* impact of MCMV infection on IL-33 expression, we have measured IL-33 mRNA levels at day 1.5, 4, 7 and 10 post infection in the liver of BALB/c mice. IL-33 mRNA expression peaked at day 4 post infection in the liver (Fig 4A) and correlated well with the kinetics of viral replication in this organ and subsequent hepatic inflammation [34, 35], confirming its role as an alarmin. In an attempt to characterize the cellular source of IL-33 we performed immunohistological co-staining of IL-33 and MCMV IE1 in liver tissue from uninfected and infected BALB/c mice. In livers of uninfected mice, IL-33 has been demonstrated in a small number of sinusoidal endothelial cells, but not in hepatocytes. In MCMV infected livers, IL-33 was found to be concentrated in a large number of cells that form focal infiltrates by selectively surrounding the foci of infection (Fig 4B). To identify cell type that produces IL-33 in these inflammatory foci of liver tissue, 2-color immunohistochemical (2C-IHC) staining was performed using antibodies to CD31 for endothelial cells (EC), CD3ε for T and NKT cells, or F4/80 (Ly71) for macrophages (Mø). As shown in Fig 5, only cells expressing F4/80 co-localized with IL-33 (Fig 5A) and indeed co-expressed IL-33 (Fig 5B), thus demonstrating that macrophages are the major IL-33 producing cells in the liver of MCMV infected mice.

### ST2 signaling is essential for Treg accumulation and the prevention of liver damage during MCMV infection

**Fig 6:**
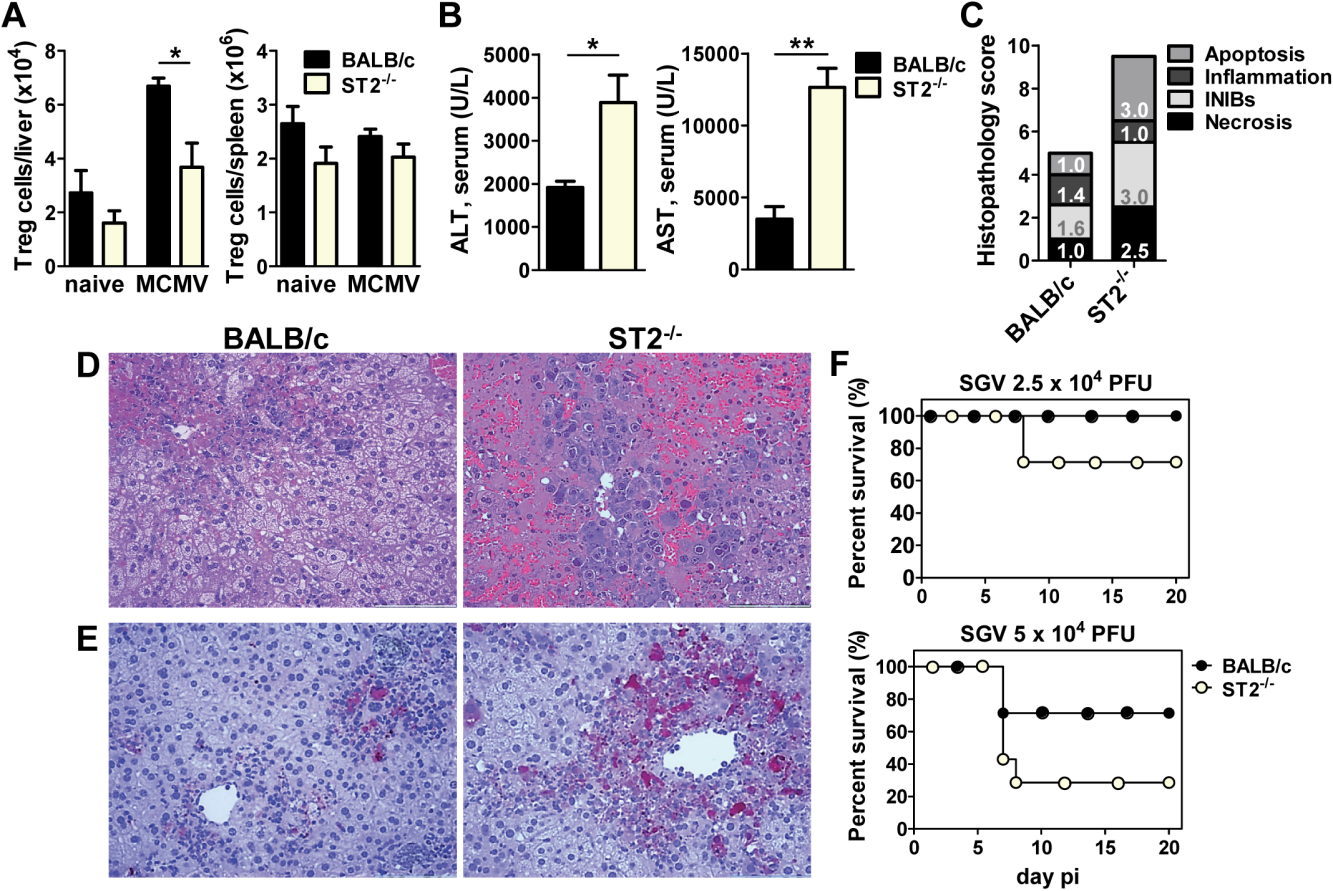
ST2^-/-^ mice show increased liver damage and mortality rate after MCMV infection. (**A**) BALB/c and ST2^-/-^ mice were i.v. injected with 2x10^5^ PFU of WT MCMV (MW97.01) and lymphocytes from spleen and liver were analyzed on day 7 p.i. Absolute number of Treg cells is shown. (**B**-**E**) BALB/c and ST2^-/-^ mice were i.v. injected with 10^6^ PFU of WT MCMV (pSM3fr-MCK-2fl clone 3.3) and analyzed on day 5 p.i. (**B**) AST and ALT serum levels were determined. (**C**) Scores of cumulative liver pathology for apoptosis, intranuclear inclusion bodies (INIBs), inflammation, and necrosis. Bars correspond to the mean score for each parameter. The height of each bar represents the mean of the total histological score (out of 12). (**D**) Representative H&E and (**E**) Caspase-3 staining of paraffin embedded liver sections. (**F**) BALB/c and ST2^-/-^ mice were i.p. injected with indicated doses of SGV MCMV. Survival rates were monitored daily. Data are shown as mean ± SEM of n = 3–5 mice from one representative experiment out of three. For survival monitoring n = 7. *p <0.05 and **p<0.01 from two tailed, unpaired Student’s t-test.

To further test whether IL-33 is important for the Treg cell response during MCMV infection we have used ST2-deficient (ST2^-/-^) mice. WT and ST2^-/-^ mice were infected with MCMV and analyzed on day 7 p.i. The accumulation of Treg cells in the liver of ST2^-/-^ mice was significantly impaired during the peak of the Treg cell response, compared to their WT counterparts (Fig 6A). In line with a reduction in the accumulation of liver Treg cells in ST2^-/-^ mice, we have detected 2-3-fold higher AST and ALT levels in ST2^-/-^ mice compared to WT mice infected with MCMV (Fig 6B). Similar to finding in Treg depleted MCMV infected mice, a number of distinct presentations of liver pathology were observed histologically in liver of ST2^-/-^ mice characterized by confluent areas of hepatocellular necrosis, mononuclear infiltrates and extravasation of erythrocytes (Fig 6C and 6D). In Con A induced hepatitis, IL-33 has been reported to suppress the expression of active caspase-3 in the liver parenchyma leading to cell apoptosis [36]. We have also investigated whether IL-33 affects the level of expression of caspase-3 in the liver of MCMV infected mice. The number of caspase-3 positive cells was remarkably higher in the liver of MCMV infected ST2^-/-^ mice compared to WT mice, indicating a protective role of IL-33/ST2 signaling in MCMV induced apoptosis and liver injury (Fig 6E). Next, we have studied whether the differences in liver damage could influence the survival rate of ST2^-/-^ mice after the infection with a highly virulent salivary gland-derived MCMV (SGV). All of the BALB/c mice survived infection with 2.5x10^4^ PFU of the SGV and resisted a dose of 5x10^4^ PFU of the SGV better than ST2^-/-^ mice (Fig 6F). Thus, the absence of IL-33 signaling leads to a reduced accumulation of Treg cells in the liver and consequently a more severe liver pathology in MCMV infected mice.

**Fig 7:**
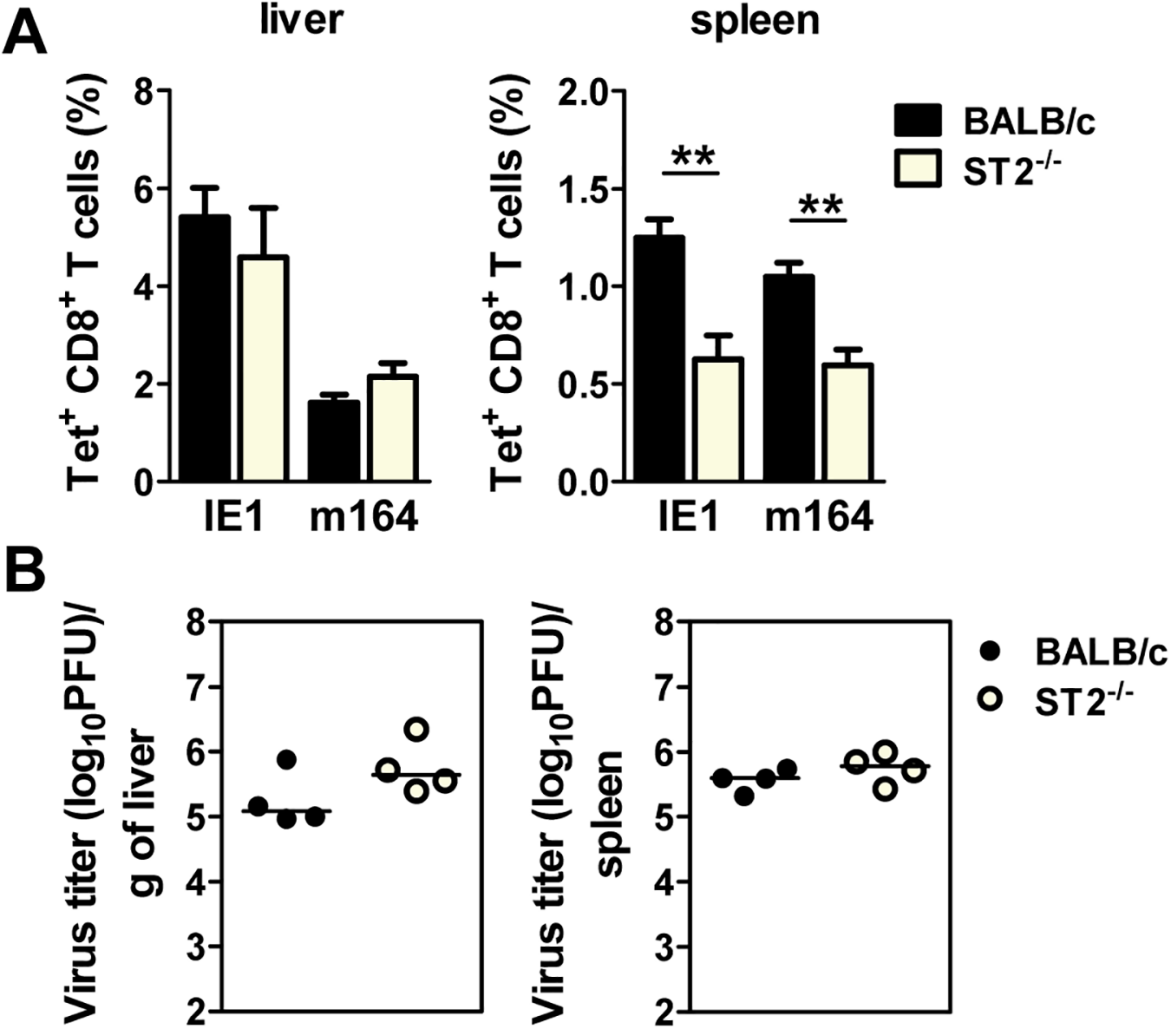
ST2 deficiency does not affect viral control. (**A**) WT and ST2^-/-^ mice were injected i.v. with 2x10^5^ PFU of WT MCMV (MW97.01) and lymphocytes from the spleen and liver were isolated on day 4 p.i. The percentages of IE1-specific and m164-specific CD8^+^ T are shown. Data are shown as mean ± SEM. (**B**) WT and ST2^-/-^ mice were injected i.v. with 10^6^ PFU of WT MCMV (pSM3fr-MCK-2fl clone 3.3). Viral titers in indicated organs 5 days post infection were determined by the plaque assay. A circle depicts the titer for each individual mouse; a small horizontal line indicates the median. n = 4–5 mice from one representative experiment out of three. **p<0.01 from two tailed, unpaired Student’s t-test.

We next assessed the impact of ST2 deficiency on CD8^+^ T cell responses to MCMV. We analyzed these effector cells in the spleen and liver of WT and ST2^-/-^ mice. In agreement with a previously published study [32] the absence of ST2 affected the antiviral CD8^+^ T cell response. Specifically, ST2^-/-^ mice showed a reduced frequency of CD8^+^ T cells directed to the immunodominant MCMV epitopes IE1/m123 and m164 in spleen. However, ST2^-/-^ mice had a comparable frequency of virus-specific CD8^+^ T cells in the liver as their WT counterparts, suggesting that the accumulation of these cells in the liver is taking place in spite of their lower frequency in lymphoid organs (Fig 7A). Thus, the impaired CD8^+^ T cell response is likely an intrinsic function of ST2 deficiency rather than effect of this pathway on Treg cells. Notably, the difference in CD8^+^ T cell response in the spleen did not affect the virus control, as ST2-deficient mice were able to control MCMV with the same efficiency as WT mice (Fig 7B). Similarly to the above, infection of mice with SGV resulted in no difference between WT and ST2-deficient mice (S6 Fig). This is in line with our earlier studies which have shown that even a complete lack of CD8^+^ T cells did not impair the kinetics of virus clearance during primary infection [37].

### Intrinsic requirement for ST2 expression in Treg cells

**Fig 8:**
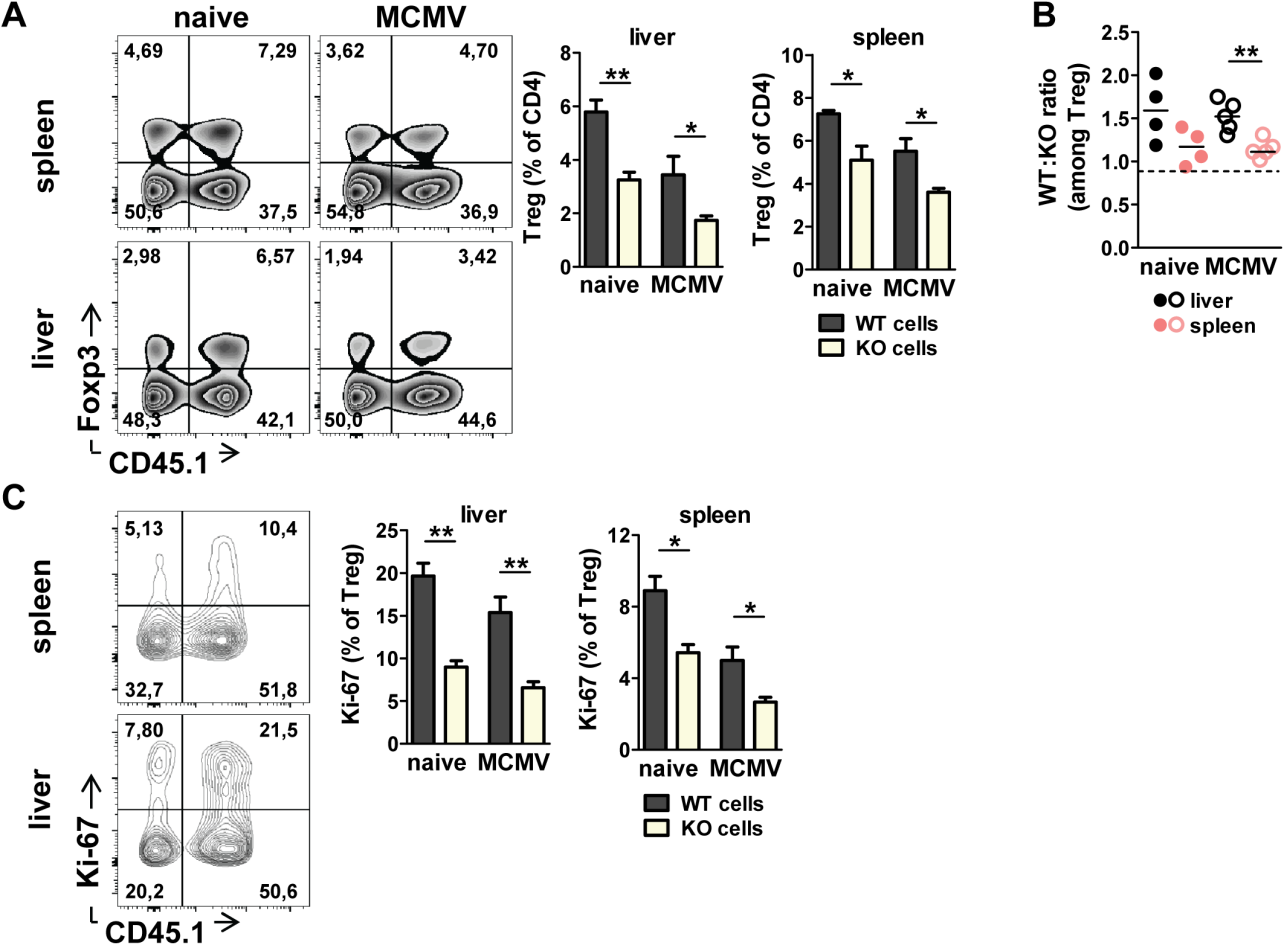
Intrinsic requirement for ST2 expression in Treg cells. Mixed bone marrow chimeras were generated by irradiation of C57BL/6 CD45.1^+^CD45.2^+^ mice followed by i.v. injection of 1:1 mixture of wild-type (WT; CD45.1^+^) and knockout (ST2^-/-^; CD45.2^+^) bone marrow cells. After reconstitution, mixed chimeras were infected with 2x10^5^ PFU of Δm157 MCMV and analyzed on day 7 p.i. (**A**) Percentage of donor WT and KO Treg cells is shown. (**B**) The ratio between WT and KO Treg cells is shown. Dotted line represents initial ratio of transferred bone marrow cells. (**C**) Percentage of Ki-67 expression by donor Treg cells is shown. Data are shown as mean ± SEM of n = 4–5 mice per group from one representative experiment out of two. *p <0.05 and **p<0.01 from two tailed, unpaired Student’s t-test.

To test whether the requirement for ST2 expression *in vivo* was Treg cell intrinsic we approached the model of mixed bone marrow chimeras generated from congenically marked CD45.1^+^ WT and CD45.2^+^ ST2^-/-^ hematopoietic cells. Chimeras were infected with Δm157 MCMV to avoid dominant NK cell recognition via Ly49H/m157 axis in mice on C57BL/6 genetic background. Analysis of splenic and liver Treg cells showed a reduction in the proportion of ST2-deficient Treg cells in both naive and infected chimeric mice (Fig 8A). However, the ratio between WT and ST2^-/-^ Treg cells was significantly higher in the liver compared to the spleen after the MCMV infection (Fig 8B). The Treg cell intrinsic effect of ST2 signaling was also supported by a lower percentage of Ki-67 expression in ST2-deficient Treg cells compared to WT Treg cells (Fig 8C).

### Expansion of liver Treg cells by recombinant IL-33 administration

**Fig 9:**
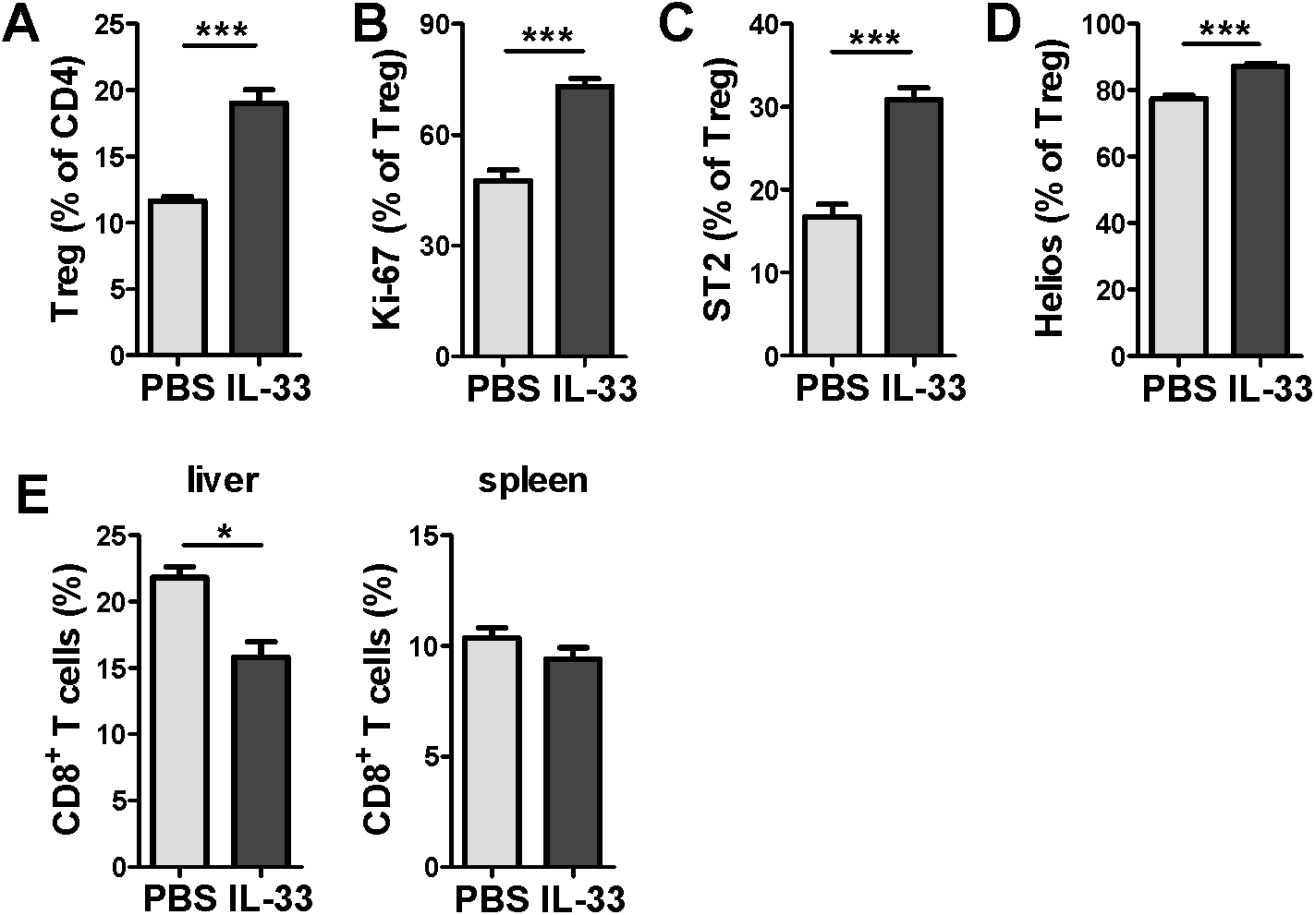
Administration of IL-33 induces liver Treg expansion. BALB/c mice were i.v. injected with 2x10^5^ PFU of WT MCMV (MW97.01) and treated i.p. with recombinant IL-33 (or PBS) on day 0 and 2 and analyzed 5 days later. (**A**) Percentage of Treg cells was determined in the liver. Percentage of (**B**) Ki-67, (**C**) ST2 and (**D**) Helios by liver Treg cells. (**E**) Percentage of CD8^+^ T cells (among live lymphocytes) was determined in the spleen and liver. Data are shown as mean ± SEM of n = 5 mice from one representative experiment out of three. *p <0.05 and ***p<0.001 from two tailed, unpaired Student’s t-test.

Next, we have analyzed whether the treatment of infected mice with recombinant IL-33 could further boost liver Treg cells in MCMV infected mice. BALB/c mice were injected with IL-33 or vehicle (phosphate-buffered saline; PBS) on the day of the infection with MCMV and 2 days later. On day 5 p.i. the percentage of liver Treg cells (Fig 9A), their expression of Ki-67 (Fig 9B), ST2 (Fig 9C) and Helios (Fig 9D) were all significantly increased following treatment with IL-33 compared to PBS-treated mice. Moreover, the percentage of overall CD8^+^ T cell compartment was significantly decreased in the liver after IL-33 treatment (Fig 9E). No difference was observed in the frequency of CD8^+^ T cells in spleen. These data demonstrate a strong impact of IL-33 on the Treg cell population in the liver upon MCMV infection.

## Discussion

Treg cells control immune responses under both physiological and pathological conditions. Although the role of Treg cells in homeostasis of immune response and the prevention of immunopathology is well established in different disease models, less is known about their role in the prevention of immunopathology during various viral infections. Many viruses, particularly herpes viruses, encode numerous genes aimed at suppressing the immune response; by doing so they may also prevent immunopathology [38]. CMV is widely spread among mammalian hosts and is usually well controlled by the immune system. However, CMVs establish lifelong persistent (latent) infection from which reactivation can occur whenever the immune response is compromised. Here we show that Treg cells are essential in limiting liver damage during the immune response to primary CMV infection. Liver Treg cells expand 2-3-fold and upregulate their activation markers upon MCMV infection. Ablation of Treg cells in MCMV infected mice led to a significant increase in liver pathology and consequent body weight loss. Liver pathology in Treg depleted mice correlated with an enhanced CD8^+^ T cell response but not with virus load. Similar to previous studies, no significant difference of virus titers was observed between Treg depleted and non-depleted mice or mice in which Treg cells were selectively expanded to avoid graft-versus-host disease [16, 39]. We have shown that CD8^+^ T cells readily accumulate in liver of CMV infected mice and are heavily activated, and that a significant proportion of them express granzyme B and IFNγ. It has been well established that the T cell response to viral infections contributes not only to limit viral replication but can also cause immunopathology if the response is not properly regulated [40]. Primary infection with non-cytolytic viruses such as hepatitis C virus (HCV) and hepatitis B virus (HBV), results in T cell mediated immunopathology in the liver [8, 41]. Being a cytolytic virus, CMV is not a prototype of virus that induces immunopathology in the immunocompetent host. Yet, recently evidence has been gathered pointing out that part of liver pathology after MCMV infection is a consequence of the immune response rather than the virus infection itself [42, 43]. The absence of liver pathology in MCMV infected SCID mice suggests the role of T cell immunity in liver damage. Here we confirm these observations and provide evidence that Treg cells dampen an exaggerated immune response and are crucial for the prevention of CMV-induced immunopathology in the liver. This is further supported by our observation of a strong proliferative capacity and activation status of liver Treg cells.

Viruses use different mechanisms to induce Treg cells. Among these, the most well known mechanisms are the ones induced via the ligation of various pathogen associated molecular pattern (PAMP) receptors which are expressed on Treg cells [44]. There are many other factors involved in triggering and expansion of Treg cells including various cytokines such as IL-2, TNFα, TGFβ and galectin molecules [8]. Since CMV infection upregulates most of the factors that can induce Treg cells, it is not unexpected that these cells are strongly expanded after infection. CMV is an ubiquitous virus which can infect almost all cell types and essentially all tissues including mucosa and glands. One can predict that Treg cells play an important role in homeostasis of the local immune response and prevention of immunopathology as has been demonstrated here for the liver.

In addition to induction by pathogens, Treg cells can also be triggered by factors released by tissue damage such as IL-33. Several studies have demonstrated that administration of IL-33 both *in vivo* and *in vitro* can induce Treg cell expansion and have highlighted the importance of IL-33 signaling in Treg cells during inflammatory conditions such as obesity-induced insulin resistance, chronic colitis, acutely injured muscles or graft-versus-host disease [22, 23, 45–48]. Here we show that liver Treg cells have a constitutively higher expression of IL-33 receptor ST2 compared to splenic Treg cells. Moreover, ST2^+^ Treg cells exhibited an increased expression of Ki-67 and incorporation of BrdU compared to ST2^-^ Treg subset, suggesting their high proliferative capacity. Given that IL-33 functions as an endogenous danger signal or alarmin in response to tissue damage [49], the upregulation of IL-33 expression after MCMV infection indicates its importance in limiting virus-induced immunopathological liver damage. A study by Nabekura et al. reported the upregulation of IL-33 mRNA in the splenic fibroblastic reticular (FRC) and lymphatic endothelial cells (LEC) but not blood endothelial cells (BEC) after MCMV infection [50]. Our results demonstrate upregulation of IL-33 mRNA in the liver tissue upon MCMV infection. Moreover, here we demonstrated that the vast majority of IL-33 producing cells in the liver of MCMV infected mice are contained within the inflammatory foci and represent F4/80^+^ macrophages. In line with this, the infection of ST2-deficient mice resulted in a reduced accumulation of Treg cells in the liver, and the liver damage was similar to the one after Treg cell depletion. Therefore, our results describe a previously unknown link between IL-33 expression and Treg cell function in the context of a viral infection.

In addition to their role in the modulation of conventional immune responses, Treg cells are important in nonimmunologic disease as tissue protective modulators of the inflammatory response after tissue damage. For instance, endogenous Treg cells strongly impact the outcome of ischemic stroke [51]. Their absence dramatically accelerates postischemic activation of inflammatory cells, and their secretion of TNFα and IFNγ is key pathogenetic factor in disease progression. Treg cells may act to dampen ischemic injury in other organs including the liver [52]. So far, there is no strong evidence that Treg depletion can influence the outcome of ischemic liver injury [53]. However, an increase in the proportion of intrahepatic Treg cells was observed in rats after treatment with some immunosuppressive agents [54]. CMV infection is frequently associated with ischemic injury most likely as a consequence of a cytokine storm [35]. This is particularly obvious in case of mice infected with a highly virulent MCMV isolate derived from salivary glands. In this and previous studies we have noticed that many lesions in liver of CMV infected mice are located in the proximity of the central vein, resembling the lesions caused by ischemic injury. Thus, it is likely that the Treg response in liver of CMV-infected mice is not only induced by infection itself but also by tissue lesions that are indirect to infection.

In summary, our study demonstrates that Treg cells inhibit a severe MCMV-induced immunopathological hepatitis. This function of Treg cells is largely dependent on IL-33-induced accumulation of Treg cells. Overall, The IL-33/Treg axis seems to be a promising route for the development of future therapies in viral infections.

## Methods

### Mice and viruses

BALB/c, DEREG BALB/c [29], ST2^-/-^ BALB/c [55], ST2^-/-^ C57BL/6 [55] kindly provided by Daniel Pinschewer, CBySmn.CB17-Prkdcscid/J (SCID), C57BL/6 CD45.1^+^ and C57BL/6 CD45.1^+^CD45.2^+^ mice were housed and bred under specific-pathogen-free conditions at the Central Animal Facility of the Medical Faculty, University of Rijeka. For depletion of Foxp3^+^ Treg cells in DEREG mice, 25 ng/g body weight of DT (Merck) were injected i.p. on day of the MCMV infection and the following one. Gender- and aged- matched littermates were used as controls. To deplete Treg cells with antibodies, BALB/c mice were injected i.p. with 150 μg of anti-CD25 antibody (PC-61.5.3; BioXCell) two days before infection. TGFβ blockade was performed by i.p. injection of 500 μg of anti-TGFβ antibody (1D11.16.8; BioXCell) on the day of infection. Depletion of CD4^+^ and CD8^+^ T cells was performed by i.p. injection of 150 μg of anti-CD8 antibody (YTS 169.4) and anti-CD4 antibody (YTS 191.1), respectively, on the day of infection. IL-33 treatments were performed by i.p. injection of recombinant mouse IL-33 (Biolegend). Mice received 2 μg IL-33 at day 0 and 2 after infection. Mice were inoculated i.v. with 10^6^ plaque-forming units (PFU) of tissue culture (TC)-grown virus, except for flow cytometric and 2C-IHC analysis where the inoculum was reduced to 2x10^5^ PFU and 5x10^5^ PFU, respectively. MCMV strain MW97.01 [56] and pSM3fr-MCK-2fl clone 3.3 [57] are referred to as WT MCMV. In addition to tissue culture grown virus, for some experiments we have used salivary gland derived virus (SGV), and mutant viruses lacking *m157* [58] or *m138* gene [59]. The viral titers in organs were quantified by the standard plaque assay, as described previously [60]. Eight- to 12-week-old mice were used in all experiments. All animal experiments described in this paper were performed in accordance with the guidelines contained in the International Guiding Principles for Biomedical Research Involving Animals and approved by the Animal Welfare Committee at the University of Rijeka.

### Flow cytometry and intracellular cytokine staining

Single-cell suspensions of spleen and liver were prepared according to standard protocols. Flow cytometric analysis were performed by using anti-mouse CD4 (GK1.5), Foxp3 (FJK-16a), CD25 (PC61.5), ST2 (RMST2-2), Ki-67 (SolA15), Bcl-2 (10C4), CD69 (H1.2F3), GITR (DTA-1), CTLA-4 (UC10-4F10-11), OX40 (OX-86), CD62L (MEL-14), CD103 (2E7), LAP (TW7-16B4), GARP (YGIC86), IL-10 (JES5-16E3), granzyme B (NGZB), CD8 (53–6.7), IFNγ (XMG1.2), KLRG1 (2F1), PD-1 (J43), Helios (22F6), Neuropilin-1 (3DS304M), IL-33 (396118), CD45.1 (A20) and CD45.2 (104) purchased from eBioscience or R&D Systems. Intracellular staining for Foxp3, IL-10, IFNγ, IL-33, Ki-67, Bcl-2 and granzyme B was performed with the Foxp3 staining kit from eBioscience according to the manufacturer’s recommendations. For the cell proliferation assay, mice were provided for 6 days with 0.8 mg/ml BrdU in the drinking water starting on the day of infection (BrdU; Sigma) To detect incorporated BrdU, cells were stained according to the manufacturer’s protocol (BrdU flow kit; BD Pharmingen). Fixable Viability Dye from eBioscience was used to stain dead cells. For intracellular cytokine staining, cells were re-stimulated with plate-bound anti-CD3 (5 μg/ml per well; 145-2C11; eBioscience) and anti-CD28 (2 μg/ml per well; 37.51; eBioscience) for 4h in the presence of Brefeldin A (10 μg/ml; eBioscience). For IFNγ staining, cells were re-stimulated with 1 μg of peptides IE1/m123 (168–176 YPHFMPTNL176) or m164 (257–265 AGPPRYSRI265) for 4h in the presence of Brefeldin A (10 μg/ml; eBioscience). H-2L(d)/IE-1 (168–176 YPHFMPTNL) and H-2D(d)/m164 (257–265 AGPPRYSRI) tetramers were provided by NIH tetramer core facility. All data were acquired using FACSAria or FACSVerse (BD Biosciences) and analyzed using FlowJo software (Tree Star).

### Adoptive transfer

Single-cell suspensions of spleen used in adoptive transfers were prepared according to standard protocols. Splenic Treg cells were isolated using CD4^+^CD25^+^ Regulatory T Cell Isolation Kit (Miltenyi Biotec). Splenic CD8^+^ T cells were isolated using CD8a^+^ T cell isolation kit (Miltenyi Biotec). A total of 1x10^7^ conventional T cells or 2x10^6^ CD8^+^ T cells and 1x10^6^ Treg cells in a total volume of 500μl of DMEM were injected into tail veins of SCID mice. Mice were injected with MCMV 8 hours before the adoptive transfer.

### Immunohistochemical (IHC) analyses of viral and cellular protein expression

Formalin-fixed, paraffin-embedded liver sections were used for hematoxylin and eosin staining and Caspase-3 (Asp175; Cell Signaling Technology) immunohistochemical staining. For the identification of the IL33-expressing mononuclear infiltrate cell type that surrounds infected hepatocytes, thus forming foci, 2-color immunohistochemical (2C-IHC) analyses were performed on paraffin-embedded liver tissue specimens. Consecutive serial 1-μm sections were prepared for combining intranuclear IE1-specific IHC labeling of infected cells [61, 62] with the detection of cell type-specific markers, which were alternatively CD31 for endothelial cells (EC), CD3ε for T and NKT cells, and F4/80 (Ly71) for macrophages (Mø) [63], as well as with the detection of IL33. For demasking antigens, the method of heat-induced epitope retrieval (HIER) [61] was employed for CD31 and CD3ε IHCs with EDTA buffer (10 mM; pH 8.0) or for the IL33 IHC with tri-sodium-citrate-dihydrate buffer (10 mM; pH 6.0). F4/80 IHC does not require HIER. In the first step, specific labeling was performed alternatively with antibodies directed against CD31 (dianova, clone SZ31), CD3ε (BioRad, clone CD3-12), F4/80 (eBioscience, clone BM8), or IL33 (Bioss Antibodies, polyclonal rabbit antiserum). For CD31, CD3ε, and F4/80 IHCs, biotin-conjugated polyclonal anti-rat Ig antibody (BD Biosciences) was used as the secondary antibody, and black staining was achieved with peroxidase-coupled avidin biotin complex (Vectastain Elite ABC Kit) using DAB as the substrate and ammonium nickel sulfate hexahydrate for color enhancement [61]. In the case of IL33 IHC, the secondary antibody was biotin-conjugated polyclonal goat aniserum anti-rabbit-IgG (Sigma-Aldrich), and turquoise-green staining was achieved with peroxidase-coupled avidin biotin complex (Vectastain Elite ABC Kit) as described above, except that substrate and chromogen were provided by the HRP-Green Solution Set (42 life sciences). Alternatively, black staining was achieved with DAB as the substrate followed by color-enhancement as described above. Finally, viral IE1 protein in the nuclei of infected cells, (of infected hepatocytes in the specific case) has been labeled with monoclonal antibody CROMA 101 [35], and red staining was achieved with alkaline phosphatase-conjugated polyclonal goat anti-mouse IgG (BioRad) and the Fuchsin^+^ substrate-chromogen system (Dako). For 2C-IHC of macrophages coexpressing F4/80 and IL33, HIER was performed for demasking IL33 epitopes, followed by turquoise-green staining as described above. After this, the macrophage marker F4/80 was stained in red after specific labeling with monoclonal rat antibody anti-F4/80 (eBioscience, clone BM8), followed by alkaline phosphatase-conjugated anti-rat Ig antibodies (BioRad, polyclonal goat serum) and the Fuchsin^+^ substrate-chromogen system.

Slides were analyzed on a Zeiss Axiophot 1 or Olympus BX51 microscope, and digital images were acquired by the VisiCAM-100 Imaging System (Visitron) using a CCD camera (Basler) or Olympus camera (DP71).

### Evaluation of liver pathology

Scores of cumulative liver pathology for apoptosis, intranuclear inclusion bodies (INIBs), inflammation, and necrosis were determined using the following scoring system: 0, normal (no pathology); 1, mild (1–3 abnormal areas); 2, moderate (3 to 5 abnormal areas); 3, severe (>5 abnormal areas) [42, 64]. Histological samples were blinded prior to evaluation. The presence of aspartate aminotransferase (AST) and alanine aminotransferase (ALT) in previously frozen serum samples was determined by standard enzymological methods in the Clinical Institute of Laboratory Diagnostics, Rijeka Clinical Hospital Center or LABOKLIN GmbH&Co.KG, Linz.

### Cell lines

B12 and RAW 264.7 cells were grown in DMEM supplemented with 10% FCS. BALB/c LSECs were grown in RPMI supplemented with 10% FCS. Cell were infected with 3 PFU/cell of Δm138 MCMV, harvested 24 hours later and stained for intracellular IL-33 expression.

### RNA extraction and real-time quantitative PCR

Total RNA was isolated from liver tissue with High Pure RNA Tissue Kit (Roche) and reversely transcribed to cDNA with High-Capacity cDNA Reverse Transcription Kit (Applied Biosystems). Real-time PCR was performed using TaqMan assay (Mm00505403_m1; Applied Biosystems). Values were normalized to mouse GAPDH (Mm99999915_g1).

### Bone marrow chimeras

Mixed bone marrow chimeras were generated by lethal irradiation (9.5 Gy) of C57BL/6 CD45.1^+^CD45.2^+^ mice followed by i.v. injection of 5x10^6^ wild-type (WT; CD45.1^+^) and 5x10^6^ ST2^-/-^ (CD45.2^+^) bone marrow cells. Mice were allowed 8 weeks to reconstitute. After reconstitution, mixed chimeras were i.v. injected with Δm157 MCMV. The analysis was performed 7 days later.

### Statistical analyses

Unless otherwise noted, data are presented as mean ± SEM. Statistical significance was determined by either two-tailed unpaired Student’s t test or one-way ANOVA with Bonferroni correction. Differences in viral titers between experimental groups were determined by the unpaired two-tailed Mann-Whitney u test using GraphPad Prism 5. A value of p>0.05 was deemed not statistically significant (ns); *p<0.05, **p<0.01 and ***p<0.001.

### Ethics statement

The study has been approved by the Animal Welfare Committee at the University of Rijeka.

## Supporting information

S1 Fig MCMV infection induces liver Treg cells. BALB/c mice were i.v. injected with 2x10^5^ PFU of WT MCMV (clone MW97.01) or left uninfected. (**A**) Percentage of Treg cells (among live lymphocytes) in spleen and liver is shown. (**B**) Graphs show expression of Helios by Ki-67^+^ Treg cells from uninfected and 7 days infected mice. (**C**) Representative FACS plots showing the surface LAP and GARP expression or intracellular IL-10 and granzyme B (Gzm B) expression of live CD4^+^ T cells after CD3/CD28 *ex vivo* restimulation. (**D**) Absolute number of ST2^+^ Treg cells in spleen and liver is shown.
(TIF)

S2 Fig ST2^+^ Treg cells in the liver exhibit an activated phenotype. (**A**) The histograms represent the intracellular expression of Ki-67 and Bcl-2 by splenic ST2^+^ (red) and ST2^-^ (blue) Treg cells (**B**) BALB/c mice were i.v. injected with 2x10^5^ PFU of WT MCMV (clone MW97.01) or left uninfected. Mice were treated with BrdU in drinking water for 6 days starting at the day of infection. Percentage of BrdU incorporation by ST2^+^ (red) and ST2^-^ (blue) Treg cells on day 7 was determined. (**C**) Representative FACS plots showing the intracellular expression of Helios and surface expression of Neuropilin-1 on Treg cells. (**D**) Histograms show representative expression of different markers by ST2^+^ and ST2^-^ liver Treg cells. (**E**) BALB/c mice were i.v. injected with 2x10^5^ PFU of WT MCMV (clone MW97.01) or left uninfected and analyzed 7 days later. Graphs show the median fluorescence intensity (MFI) of expression of different markers by liver ST2^+^ and ST2^-^ Treg cells. Data are shown as mean ± SEM of n = 3–5 mice from one representative experiment out of three. *p<0.05; **p<0.01; ***p<0.001 from two tailed, unpaired Student’s t-test.
(TIF)

S3 Fig Anti-CD25 treatment results in liver damage upon MCMV infection. BALB/c mice were infected with 10^6^ PFU of WT MCMV and treated with anti-CD25. (**A**) Mice were analyzed on day 5 p.i. and serum AST and ALT were determined. (**B**) Viral titers in indicated organs on day 5 p.i. (**C**) Naive BALB/c DEREG mice were treated i.p. with DT on day 0 and 1 or left untreated. AST and ALT levels were determined in the serum 5 days later. Data are shown as mean ± SEM of n = 4–5 mice from one representative experiment out of two. *p <0.05 from two tailed, unpaired Student’s t-test.
(TIF)

S4 Fig Treg depletion results in liver immunopathology mediated by CD4^+^ and CD8^+^ T cells in MCMV infected mice. BALB/c DEREG mice were i.p. injected with either anti-TGFβ, anti-CD8 or anti-CD4 antibody 3 hours prior to infection. Mice were i.v. injected with 10^6^ WT MCMV (pSM3fr-MCK-2fl clone 3.3) and treated i.p. with DT on day 0 and 1 or left untreated. Mice were analyzed on day 5 p.i. (**A**) AST and ALT levels were determined in the serum. Pooled data from 2 independent experiments are shown as mean ± SEM of n = 8–9 mice (**B**) Changes in the body weight on day 4 p.i. were determined as a percent of weight at the date of infection. Data are shown as mean ± SEM of n = 5–6 mice from one representative experiment. (**C**) BALB/c SCID mice were i.v. injected with 10^6^ WT MCMV (pSM3fr-MCK-2fl clone 3.3) and at the same day of infection received 2x10^6^ CD8^+^ T cells from naive BALB/c mice alone or together with 1x10^6^ Treg cells. ALT levels were determined in the serum on day 5 p.i. Data are shown as mean ± SEM of n = 3–4 mice from one representative experiment. *p<0.05; **p<0.01; ***p<0.001 from two tailed, unpaired Student’s t-test.
(TIF)

S5 Fig IL-33 expression is increased during MCMV infection *in vitro*. B12, RAW 264.7 and LSEC cells were infected with Δm138 MCMV or left uninfected. Cells were harvested 24 hours p.i. and stained for intracellular IL-33. Data are representative of three independent experiments.
(TIF)

S6 Fig Viral titers in the spleen and liver of BALB/c and ST2^-/-^ mice after SGV infection. BALB/c and ST2^-/-^ mice were i.p. injected with 5x10^4^ PFU of SGV MCMV. Viral titers in indicated organs 6 days post infection were determined by the plaque assay. A circle depicts the titer for each individual mouse; a small horizontal line indicates the median. n = 5 mice from one representative experiment out of two.
(TIF)

## References

[1] Josefowicz SZ, Lu LF, Rudensky AY. Regulatory T cells: mechanisms of differentiation and function. Annu Rev Immunol. 2012;30:531–64. doi: 10.1146/annurev.immunol.25.022106.141623 22224781 10.1146/annurev.immunol.25.022106.141623 PMC6066374

[2] Curotto de Lafaille MA, Lafaille JJ. Natural and adaptive foxp3+ regulatory T cells: more of the same or a division of labor? Immunity. 2009;30(5):626–35. doi: 10.1016/j.immuni.2009.05.002 19464985 10.1016/j.immuni.2009.05.002

[3] Cretney E, Kallies A, Nutt SL. Differentiation and function of Foxp3(+) effector regulatory T cells. Trends Immunol. 2013;34(2):74–80. doi: 10.1016/j.it.2012.11.002 23219401 10.1016/j.it.2012.11.002

[4] Burzyn D, Benoist C, Mathis D. Regulatory T cells in nonlymphoid tissues. Nat Immunol. 2013;14(10):1007–13. PubMed Central PMCID: PMCPMC4708287. doi: 10.1038/ni.2683 24048122 10.1038/ni.2683 PMC4708287

[5] Arpaia N, Green JA, Moltedo B, Arvey A, Hemmers S, Yuan S, et al A Distinct Function of Regulatory T Cells in Tissue Protection. Cell. 2015;162(5):1078–89. PubMed Central PMCID: PMCPMC4603556. doi: 10.1016/j.cell.2015.08.021 26317471 10.1016/j.cell.2015.08.021 PMC4603556

[6] Molofsky AB, Savage AK, Locksley RM. Interleukin-33 in Tissue Homeostasis, Injury, and Inflammation. Immunity. 2015;42(6):1005–19. PubMed Central PMCID: PMCPMC4471869. doi: 10.1016/j.immuni.2015.06.006 26084021 10.1016/j.immuni.2015.06.006 PMC4471869

[7] Peine M, Marek RM, Löhning M. IL-33 in T Cell Differentiation, Function, and Immune Homeostasis. Trends Immunol. 2016. 10.1016/j.it.2016.03.007 27055914

[8] Veiga-Parga T, Sehrawat S, Rouse BT. Role of regulatory T cells during virus infection. Immunol Rev. 2013;255(1):182–96. PubMed Central PMCID: PMCPMC3748387. doi: 10.1111/imr.12085 23947355 10.1111/imr.12085 PMC3748387

[9] Lund JM, Hsing L, Pham TT, Rudensky AY. Coordination of early protective immunity to viral infection by regulatory T cells. Science. 2008;320(5880):1220–4. PubMed Central PMCID: PMCPMC2519146. doi: 10.1126/science.1155209 18436744 10.1126/science.1155209 PMC2519146

[10] Ruckwardt TJ, Bonaparte KL, Nason MC, Graham BS. Regulatory T cells promote early influx of CD8+ T cells in the lungs of respiratory syncytial virus-infected mice and diminish immunodominance disparities. J Virol. 2009;83(7):3019–28. PubMed Central PMCID: PMCPMC2655550. doi: 10.1128/JVI.00036-09 19153229 10.1128/JVI.00036-09 PMC2655550

[11] Suvas S, Kumaraguru U, Pack CD, Lee S, Rouse BT. CD4+CD25+ T cells regulate virus-specific primary and memory CD8+ T cell responses. J Exp Med. 2003;198(6):889–901. PubMed Central PMCID: PMCPMC2194203. doi: 10.1084/jem.20030171 12975455 10.1084/jem.20030171 PMC2194203

[12] Zelinskyy G, Dietze KK, Hüsecken YP, Schimmer S, Nair S, Werner T, et al The regulatory T-cell response during acute retroviral infection is locally defined and controls the magnitude and duration of the virus-specific cytotoxic T-cell response. Blood. 2009;114(15):3199–207. doi: 10.1182/blood-2009-03-208736 19671923 10.1182/blood-2009-03-208736

[13] Belkaid Y, Rouse BT. Natural regulatory T cells in infectious disease. Nat Immunol. 2005;6(4):353–60. doi: 10.1038/ni1181 15785761 10.1038/ni1181

[14] Lemmermann NA, Böhm V, Holtappels R, Reddehase MJ. In vivo impact of cytomegalovirus evasion of CD8 T-cell immunity: facts and thoughts based on murine models. Virus Res. 2011;157(2):161–74. doi: 10.1016/j.virusres.2010.09.022 20933556 10.1016/j.virusres.2010.09.022

[15] Lisnić B, Lisnić VJ, Jonjić S. NK cell interplay with cytomegaloviruses. Curr Opin Virol. 2015;15:9–18. doi: 10.1016/j.coviro.2015.07.001 26208082 10.1016/j.coviro.2015.07.001

[16] Jost NH, Abel S, Hutzler M, Sparwasser T, Zimmermann A, Roers A, et al Regulatory T cells and T-cell-derived IL-10 interfere with effective anti-cytomegalovirus immune response. Immunol Cell Biol. 2014;92(10):860–71. doi: 10.1038/icb.2014.62 25070242 10.1038/icb.2014.62

[17] Lindenberg M, Solmaz G, Puttur F, Sparwasser T. Mouse cytomegalovirus infection overrules T regulatory cell suppression on natural killer cells. Virol J. 2014;11:145 PubMed Central PMCID: PMCPMC4254395. doi: 10.1186/1743-422X-11-145 25108672 10.1186/1743-422X-11-145 PMC4254395

[18] Lokensgard JR, Schachtele SJ, Mutnal MB, Sheng WS, Prasad S, Hu S. Chronic reactive gliosis following regulatory T cell depletion during acute MCMV encephalitis. Glia. 2015. PubMed Central PMCID: PMCPMC4670295. 10.1002/glia.22868 PMC4670295 26041050

[19] Terrazzini N, Bajwa M, Vita S, Cheek E, Thomas D, Seddiki N, et al A novel cytomegalovirus-induced regulatory-type T-cell subset increases in size during older life and links virus-specific immunity to vascular pathology. J Infect Dis. 2014;209(9):1382–92. PubMed Central PMCID: PMCPMC3982844. doi: 10.1093/infdis/jit576 24203779 10.1093/infdis/jit576 PMC3982844

[20] Walton SM, Wyrsch P, Munks MW, Zimmermann A, Hengel H, Hill AB, et al The dynamics of mouse cytomegalovirus-specific CD4 T cell responses during acute and latent infection. J Immunol. 2008;181(2):1128–34. 18606665 10.4049/jimmunol.181.2.1128

[21] Shevach EM. Mechanisms of foxp3+ T regulatory cell-mediated suppression. Immunity. 2009;30(5):636–45. doi: 10.1016/j.immuni.2009.04.010 19464986 10.1016/j.immuni.2009.04.010

[22] Schiering C, Krausgruber T, Chomka A, Fröhlich A, Adelmann K, Wohlfert EA, et al The alarmin IL-33 promotes regulatory T-cell function in the intestine. Nature. 2014;513(7519):564–8. doi: 10.1038/nature13577 25043027 10.1038/nature13577 PMC4339042

[23] Matta BM, Lott JM, Mathews LR, Liu Q, Rosborough BR, Blazar BR, et al IL-33 is an unconventional Alarmin that stimulates IL-2 secretion by dendritic cells to selectively expand IL-33R/ST2+ regulatory T cells. J Immunol. 2014;193(8):4010–20. PubMed Central PMCID: PMCPMC4185240. doi: 10.4049/jimmunol.1400481 25217167 10.4049/jimmunol.1400481 PMC4185240

[24] Jones CA. Congenital cytomegalovirus infection. Curr Probl Pediatr Adolesc Health Care. 2003;33(3):70–93. doi: 10.1067/mps.2003.3 12605193 10.1067/mps.2003.3

[25] Smith MA, Brennessel DJ. Cytomegalovirus. Infect Dis Clin North Am. 1994;8(2):427–38. 8089469

[26] Krmpotic A, Bubic I, Polic B, Lucin P, Jonjic S. Pathogenesis of murine cytomegalovirus infection. Microbes Infect. 2003;5(13):1263–77. 14623023 10.1016/j.micinf.2003.09.007

[27] Kohm AP, McMahon JS, Podojil JR, Begolka WS, DeGutes M, Kasprowicz DJ, et al Cutting Edge: Anti-CD25 monoclonal antibody injection results in the functional inactivation, not depletion, of CD4+CD25+ T regulatory cells. J Immunol. 2006;176(6):3301–5. 16517695 10.4049/jimmunol.176.6.3301

[28] Couper KN, Lanthier PA, Perona-Wright G, Kummer LW, Chen W, Smiley ST, et al Anti-CD25 antibody-mediated depletion of effector T cell populations enhances susceptibility of mice to acute but not chronic Toxoplasma gondii infection. J Immunol. 2009;182(7):3985–94. PubMed Central PMCID: PMCPMC3942880. doi: 10.4049/jimmunol.0803053 19299696 10.4049/jimmunol.0803053 PMC3942880

[29] Lahl K, Loddenkemper C, Drouin C, Freyer J, Arnason J, Eberl G, et al Selective depletion of Foxp3+ regulatory T cells induces a scurfy-like disease. J Exp Med. 2007;204(1):57–63. PubMed Central PMCID: PMCPMC2118432. doi: 10.1084/jem.20061852 17200412 10.1084/jem.20061852 PMC2118432

[30] Reddehase MJ, Rothbard JB, Koszinowski UH. A pentapeptide as minimal antigenic determinant for MHC class I-restricted T lymphocytes. Nature. 1989;337(6208):651–3. doi: 10.1038/337651a0 2465495 10.1038/337651a0

[31] Holtappels R, Thomas D, Podlech J, Reddehase MJ. Two antigenic peptides from genes m123 and m164 of murine cytomegalovirus quantitatively dominate CD8 T-cell memory in the H-2d haplotype. J Virol. 2002;76(1):151–64. PubMed Central PMCID: PMCPMC135724. doi: 10.1128/JVI.76.1.151-164.2002 11739681 10.1128/JVI.76.1.151-164.2002 PMC135724

[32] Bonilla WV, Fröhlich A, Senn K, Kallert S, Fernandez M, Johnson S, et al The alarmin interleukin-33 drives protective antiviral CD8⁺ T cell responses. Science. 2012;335(6071):984–9. doi: 10.1126/science.1215418 22323740 10.1126/science.1215418

[33] Le Goffic R, Arshad MI, Rauch M, L'Helgoualc'h A, Delmas B, Piquet-Pellorce C, et al Infection with influenza virus induces IL-33 in murine lungs. Am J Respir Cell Mol Biol. 2011;45(6):1125–32. doi: 10.1165/rcmb.2010-0516OC 21642589 10.1165/rcmb.2010-0516OC

[34] Orange JS, Salazar-Mather TP, Opal SM, Biron CA. Mechanisms for virus-induced liver disease: tumor necrosis factor-mediated pathology independent of natural killer and T cells during murine cytomegalovirus infection. J Virol. 1997;71(12):9248–58. PubMed Central PMCID: PMCPMC230227. 9371583 10.1128/jvi.71.12.9248-9258.1997 PMC230227

[35] Trgovcich J, Stimac D, Polić B, Krmpotić A, Pernjak-Pugel E, Tomac J, et al Immune responses and cytokine induction in the development of severe hepatitis during acute infections with murine cytomegalovirus. Arch Virol. 2000;145(12):2601–18. 11205107 10.1007/s007050070010

[36] Volarevic V, Mitrovic M, Milovanovic M, Zelen I, Nikolic I, Mitrovic S, et al Protective role of IL-33/ST2 axis in Con A-induced hepatitis. J Hepatol. 2012;56(1):26–33. doi: 10.1016/j.jhep.2011.03.022 21703183 10.1016/j.jhep.2011.03.022

[37] Jonjić S, Pavić I, Lucin P, Rukavina D, Koszinowski UH. Efficacious control of cytomegalovirus infection after long-term depletion of CD8+ T lymphocytes. J Virol. 1990;64(11):5457–64. PubMed Central PMCID: PMCPMC248597. 1976821 10.1128/jvi.64.11.5457-5464.1990 PMC248597

[38] Tortorella D, Gewurz BE, Furman MH, Schust DJ, Ploegh HL. Viral subversion of the immune system. Annu Rev Immunol. 2000;18:861–926. doi: 10.1146/annurev.immunol.18.1.861 10837078 10.1146/annurev.immunol.18.1.861

[39] Chopra M, Biehl M, Steinfatt T, Brandl A, Kums J, Amich J, et al Exogenous TNFR2 activation protects from acute GvHD via host T reg cell expansion. J Exp Med. 2016;213(9):1881–900. Epub 2016/08/15. PubMed Central PMCID: PMCPMC4995078. doi: 10.1084/jem.20151563 27526711 10.1084/jem.20151563 PMC4995078

[40] Doherty PC, Zinkernagel RM. T-cell-mediated immunopathology in viral infections. Transplant Rev. 1974;19(0):89–120. 4601807 10.1111/j.1600-065x.1974.tb00129.x

[41] Manigold T, Racanelli V. T-cell regulation by CD4 regulatory T cells during hepatitis B and C virus infections: facts and controversies. Lancet Infect Dis. 2007;7(12):804–13. doi: 10.1016/S1473-3099(07)70289-X 18045563 10.1016/S1473-3099(07)70289-X

[42] Livingston-Rosanoff D, Daley-Bauer LP, Garcia A, McCormick AL, Huang J, Mocarski ES. Antiviral T cell response triggers cytomegalovirus hepatitis in mice. J Virol. 2012;86(23):12879–90. PubMed Central PMCID: PMCPMC3497643. doi: 10.1128/JVI.01752-12 22993151 10.1128/JVI.01752-12 PMC3497643

[43] Arapović J, Arapović M, Golemac M, Traven L, Tomac J, Rumora D, et al The specific NK cell response in concert with perforin prevents CD8(+) T cell-mediated immunopathology after mouse cytomegalovirus infection. Med Microbiol Immunol. 2015;204(3):335–44. doi: 10.1007/s00430-015-0409-y 25809566 10.1007/s00430-015-0409-y

[44] Conroy H, Marshall NA, Mills KH. TLR ligand suppression or enhancement of Treg cells? A double-edged sword in immunity to tumours. Oncogene. 2008;27(2):168–80. doi: 10.1038/sj.onc.1210910 18176598 10.1038/sj.onc.1210910

[45] Turnquist HR, Zhao Z, Rosborough BR, Liu Q, Castellaneta A, Isse K, et al IL-33 expands suppressive CD11b+ Gr-1(int) and regulatory T cells, including ST2L+ Foxp3+ cells, and mediates regulatory T cell-dependent promotion of cardiac allograft survival. J Immunol. 2011;187(9):4598–610. PubMed Central PMCID: PMCPMC3197898. doi: 10.4049/jimmunol.1100519 21949025 10.4049/jimmunol.1100519 PMC3197898

[46] Vasanthakumar A, Moro K, Xin A, Liao Y, Gloury R, Kawamoto S, et al The transcriptional regulators IRF4, BATF and IL-33 orchestrate development and maintenance of adipose tissue-resident regulatory T cells. Nat Immunol. 2015;16(3):276–85. doi: 10.1038/ni.3085 25599561 10.1038/ni.3085

[47] Kuswanto W, Burzyn D, Panduro M, Wang KK, Jang YC, Wagers AJ, et al Poor Repair of Skeletal Muscle in Aging Mice Reflects a Defect in Local, Interleukin-33-Dependent Accumulation of Regulatory T Cells. Immunity. 2016;44(2):355–67. PubMed Central PMCID: PMCPMC4764071. doi: 10.1016/j.immuni.2016.01.009 26872699 10.1016/j.immuni.2016.01.009 PMC4764071

[48] Matta BM, Reichenbach DK, Zhang X, Mathews L, Koehn BH, Dwyer GK, et al Peri-alloHCT IL-33 administration expands recipient T-regulatory cells that protect mice against acute GVHD. Blood. 2016;128(3):427–39. Epub 2016/05/24. PubMed Central PMCID: PMCPMC4957164. doi: 10.1182/blood-2015-12-684142 27222477 10.1182/blood-2015-12-684142 PMC4957164

[49] Martin NT, Martin MU. Interleukin 33 is a guardian of barriers and a local alarmin. Nat Immunol. 2016;17(2):122–31. doi: 10.1038/ni.3370 26784265 10.1038/ni.3370

[50] Nabekura T, Girard JP, Lanier LL. IL-33 receptor ST2 amplifies the expansion of NK cells and enhances host defense during mouse cytomegalovirus infection. J Immunol. 2015;194(12):5948–52. PubMed Central PMCID: PMCPMC4458425. doi: 10.4049/jimmunol.1500424 25926677 10.4049/jimmunol.1500424 PMC4458425

[51] Liesz A, Suri-Payer E, Veltkamp C, Doerr H, Sommer C, Rivest S, et al Regulatory T cells are key cerebroprotective immunomodulators in acute experimental stroke. Nat Med. 2009;15(2):192–9. doi: 10.1038/nm.1927 19169263 10.1038/nm.1927

[52] Lu L, Li G, Rao J, Pu L, Yu Y, Wang X, et al In vitro induced CD4(+)CD25(+)Foxp3(+) Tregs attenuate hepatic ischemia-reperfusion injury. Int Immunopharmacol. 2009;9(5):549–52. doi: 10.1016/j.intimp.2009.01.020 19539564 10.1016/j.intimp.2009.01.020

[53] Kuboki S, Sakai N, Tschöp J, Edwards MJ, Lentsch AB, Caldwell CC. Distinct contributions of CD4+ T cell subsets in hepatic ischemia/reperfusion injury. Am J Physiol Gastrointest Liver Physiol. 2009;296(5):G1054–9. PubMed Central PMCID: PMCPMC2696215. doi: 10.1152/ajpgi.90464.2008 19264952 10.1152/ajpgi.90464.2008 PMC2696215

[54] Arias-Diaz J, Ildefonso JA, Muñoz JJ, Zapata A, Jiménez E. Both tacrolimus and sirolimus decrease Th1/Th2 ratio, and increase regulatory T lymphocytes in the liver after ischemia/reperfusion. Lab Invest. 2009;89(4):433–45. doi: 10.1038/labinvest.2009.3 19188906 10.1038/labinvest.2009.3

[55] Townsend MJ, Fallon PG, Matthews DJ, Jolin HE, McKenzie AN. T1/ST2-deficient mice demonstrate the importance of T1/ST2 in developing primary T helper cell type 2 responses. J Exp Med. 2000;191(6):1069–76. PubMed Central PMCID: PMCPMC2193113. 10727469 10.1084/jem.191.6.1069 PMC2193113

[56] Wagner M, Jonjic S, Koszinowski UH, Messerle M. Systematic excision of vector sequences from the BAC-cloned herpesvirus genome during virus reconstitution. Journal of virology. 1999;73(8):7056–60. PubMed Central PMCID: PMC112796. 10400809 10.1128/jvi.73.8.7056-7060.1999 PMC112796

[57] Jordan S, Krause J, Prager A, Mitrovic M, Jonjic S, Koszinowski UH, et al Virus progeny of murine cytomegalovirus bacterial artificial chromosome pSM3fr show reduced growth in salivary Glands due to a fixed mutation of MCK-2. J Virol. 2011;85(19):10346–53. PubMed Central PMCID: PMCPMC3196435. doi: 10.1128/JVI.00545-11 21813614 10.1128/JVI.00545-11 PMC3196435

[58] Bubic I, Wagner M, Krmpotic A, Saulig T, Kim S, Yokoyama WM, et al Gain of virulence caused by loss of a gene in murine cytomegalovirus. Journal of virology. 2004;78(14):7536–44. PubMed Central PMCID: PMC434107. doi: 10.1128/JVI.78.14.7536-7544.2004 15220428 10.1128/JVI.78.14.7536-7544.2004 PMC434107

[59] Crnkovic-Mertens I, Messerle M, Milotic I, Szepan U, Kucic N, Krmpotic A, et al Virus attenuation after deletion of the cytomegalovirus Fc receptor gene is not due to antibody control. Journal of virology. 1998;72(2):1377–82. PubMed Central PMCID: PMC124616. 9445038 10.1128/jvi.72.2.1377-1382.1998 PMC124616

[60] Jonjic S, Krmpotic A, Arapovic J, Koszinowski UH. Dissection of the antiviral NK cell response by MCMV mutants. Methods Mol Biol. 2008;415:127–49. doi: 10.1007/978-1-59745-570-1_8 18370152 10.1007/978-1-59745-570-1_8

[61] Podlech J, Holtappels R, Grzimek NKA, Reddehase MJ. Animal models: murine cytomegalovirus In: Kaufmann SH, Kabelitz D, editors. Methods in Microbiology: Immunology of Infection. London: Academic Press; 2002 p. 493–525.

[62] Lemmermann NA, Podlech J, Seckert CK, Kropp KA, Grzimek NK, Reddehase MJ, et al CD8 T-cell immunotherapy of cytomegalovirus disease in the murine model In: Kabelitz D, Kaufmann SHE, editors. Methods in Microbiology: Immunology of Infection. London: Academic Press; 2010 p. 369–420.

[63] Lemmermann NA, Krmpotic A, Podlech J, Brizic I, Prager A, Adler H, et al Non-redundant and redundant roles of cytomegalovirus gH/gL complexes in host organ entry and intra-tissue spread. PLoS Pathog. 2015;11(2):e1004640 Epub 2015/02/06. PubMed Central PMCID: PMCPMC4450070. doi: 10.1371/journal.ppat.1004640 25659098 10.1371/journal.ppat.1004640 PMC4450070

[64] Chiossone L, Audonnet S, Chetaille B, Chasson L, Farnarier C, Berda-Haddad Y, et al Protection from inflammatory organ damage in a murine model of hemophagocytic lymphohistiocytosis using treatment with IL-18 binding protein. Front Immunol. 2012;3:239 Epub 2012/08/08. PubMed Central PMCID: PMCPMC3413989. doi: 10.3389/fimmu.2012.00239 22891066 10.3389/fimmu.2012.00239 PMC3413989

