# Supplementary figures and images for: IL-33/ST2 pathway drives regulatory T cell dependent suppression of liver damage upon cytomegalovirus infection
Source: PLoS Pathog. 2017 Apr 27;13(4):e1006345. doi: 10.1371/journal.ppat.1006345 (PMC5423658; doi:10.1371/journal.ppat.1006345)

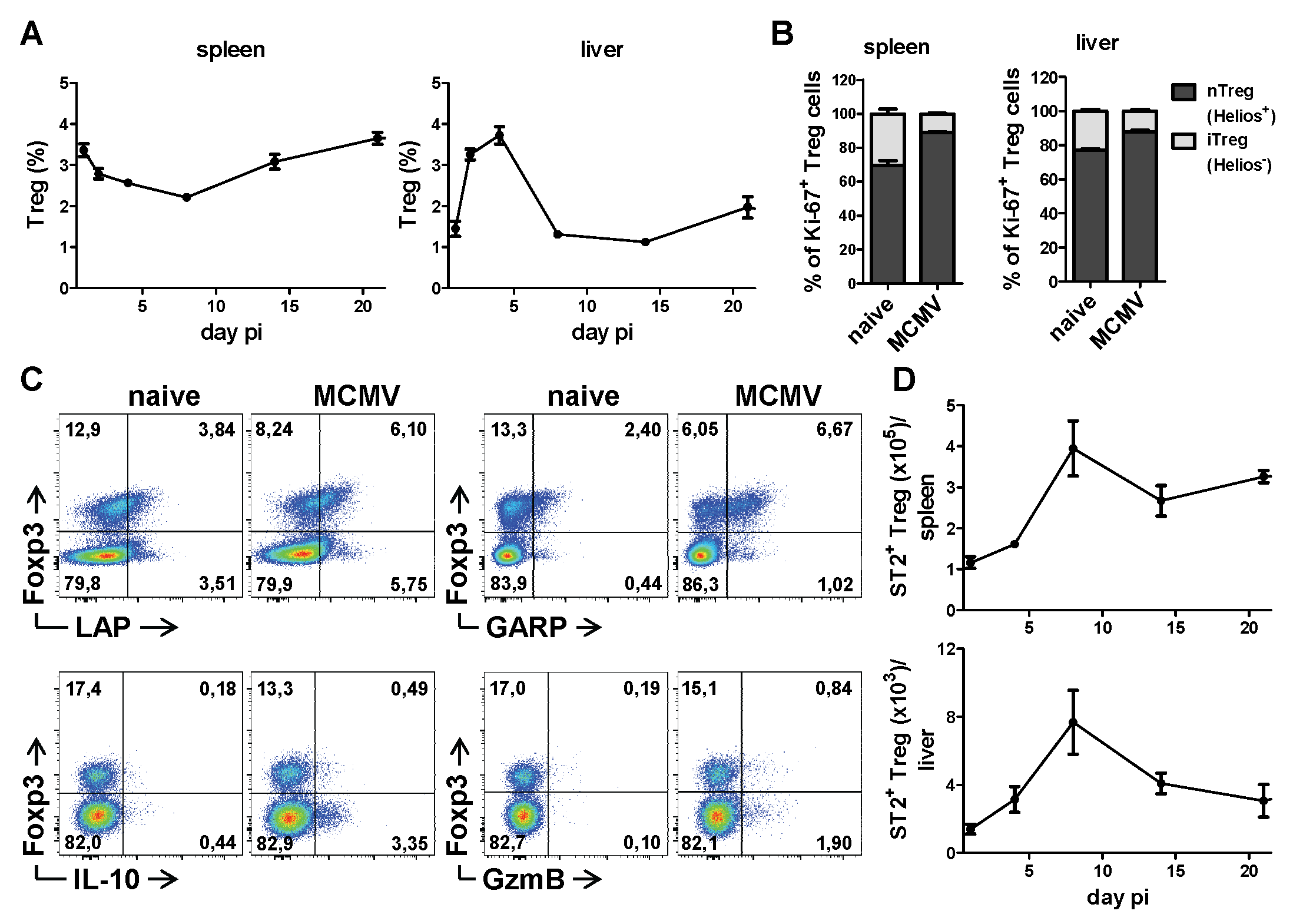

Supplement: S1 Fig — BALB/c mice were i.v. injected with 2x10^5^ PFU of WT MCMV (clone MW97.01) or left uninfected. (A) Percentage of Treg cells (among live lymphocytes) in spleen and liver is shown. (B) Graphs show expression of Helios by Ki-67+ Treg cells from uninfected and 7 days infected mice. (C) Representative FACS plots showing the surface LAP and GARP expression or intracellular IL-10 and granzyme B (Gzm B) expression of live CD4+ T cells after CD3/CD28 ex vivo restimulation. (D) Absolute number of ST2+ Treg cells in spleen and liver is shown. (TIF) [file ppat.1006345.s001.tif]

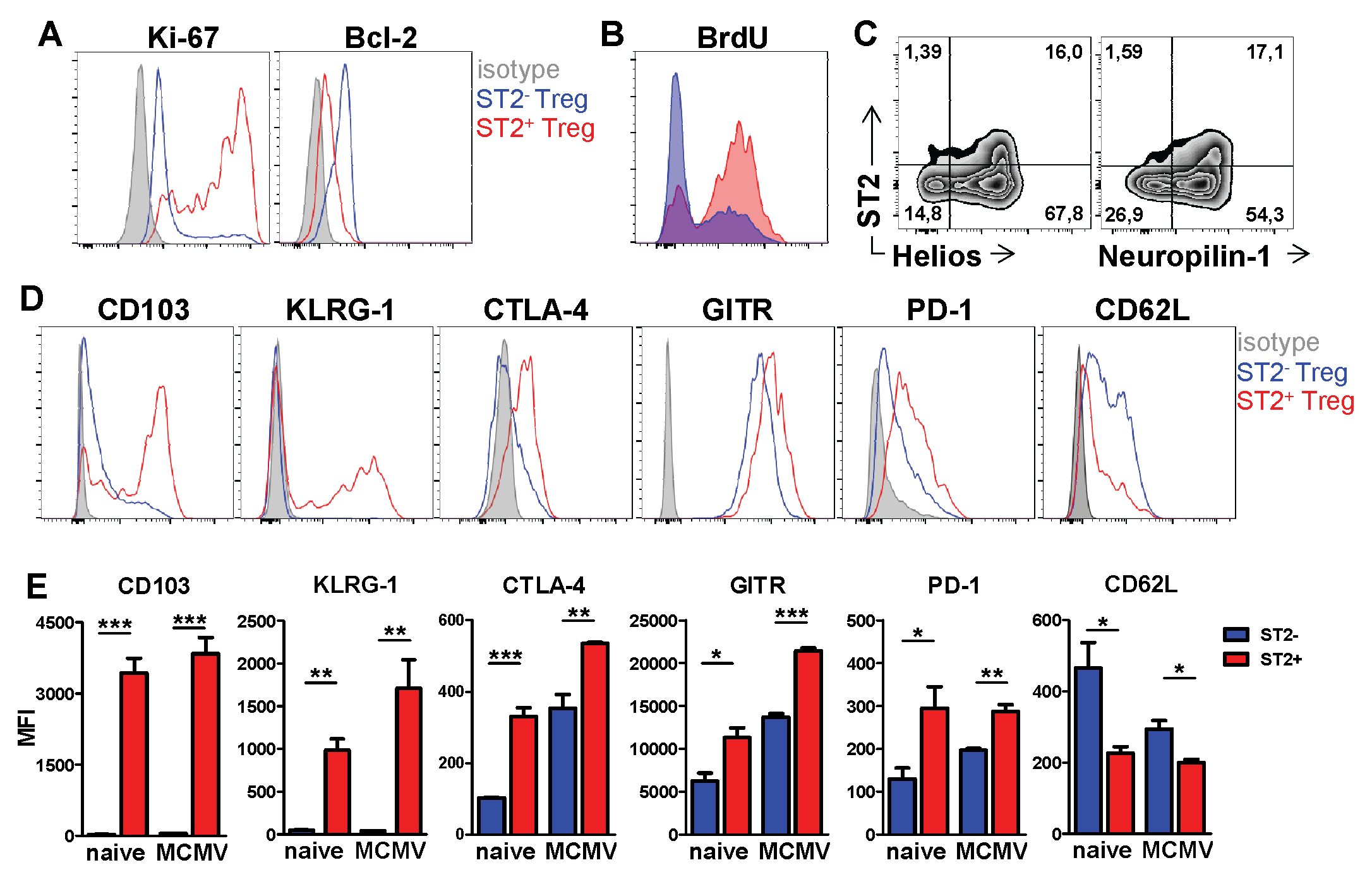

Supplement: S2 Fig — (A) The histograms represent the intracellular expression of Ki-67 and Bcl-2 by splenic ST2+ (red) and ST2- (blue) Treg cells (B) BALB/c mice were i.v. injected with 2x10^5^ PFU of WT MCMV (clone MW97.01) or left uninfected. Mice were treated with BrdU in drinking water for 6 days starting at the day of infection. Percentage of BrdU incorporation by ST2+ (red) and ST2- (blue) Treg cells on day 7 was determined. (C) Representative FACS plots showing the intracellular expression of Helios and surface expression of Neuropilin-1 on Treg cells. (D) Histograms show representative expression of different markers by ST2+ and ST2- liver Treg cells. (E) BALB/c mice were i.v. injected with 2x10^5^ PFU of WT MCMV (clone MW97.01) or left uninfected and analyzed 7 days later. Graphs show the median fluorescence intensity (MFI) of expression of different markers by liver ST2+ and ST2- Treg cells. Data are shown as mean ± SEM of n = 3–5 mice from one representative experiment out of three. *p<0.05; **p<0.01; ***p<0.001 from two tailed, unpaired Student’s t-test. (TIF) [file ppat.1006345.s002.tif]

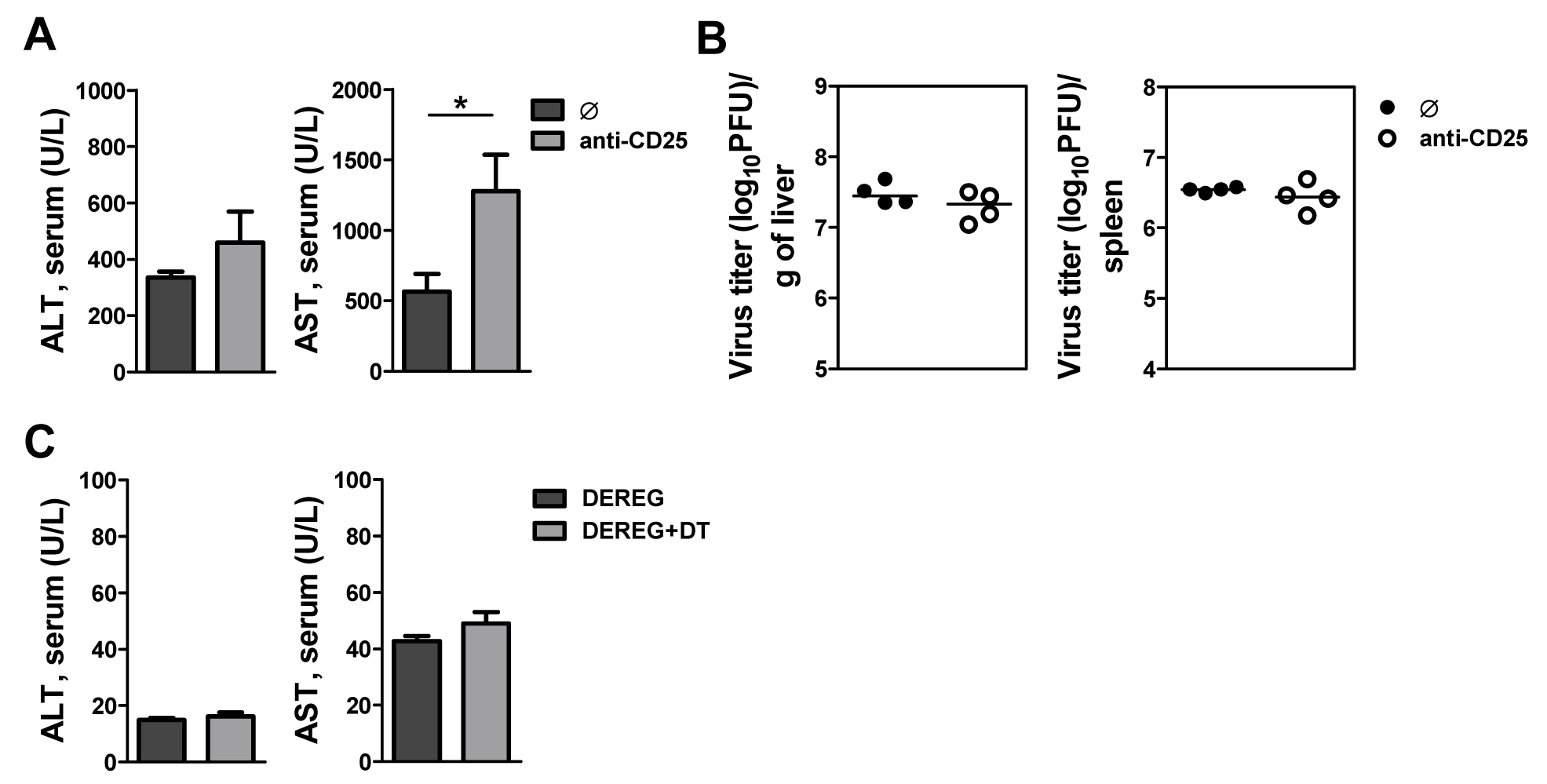

Supplement: S3 Fig — BALB/c mice were infected with 106 PFU of WT MCMV and treated with anti-CD25. (A) Mice were analyzed on day 5 p.i. and serum AST and ALT were determined. (B) Viral titers in indicated organs on day 5 p.i. (C) Naive BALB/c DEREG mice were treated i.p. with DT on day 0 and 1 or left untreated. AST and ALT levels were determined in the serum 5 days later. Data are shown as mean ± SEM of n = 4–5 mice from one representative experiment out of two. *p <0.05 from two tailed, unpaired Student’s t-test. (TIF) [file ppat.1006345.s003.tif]

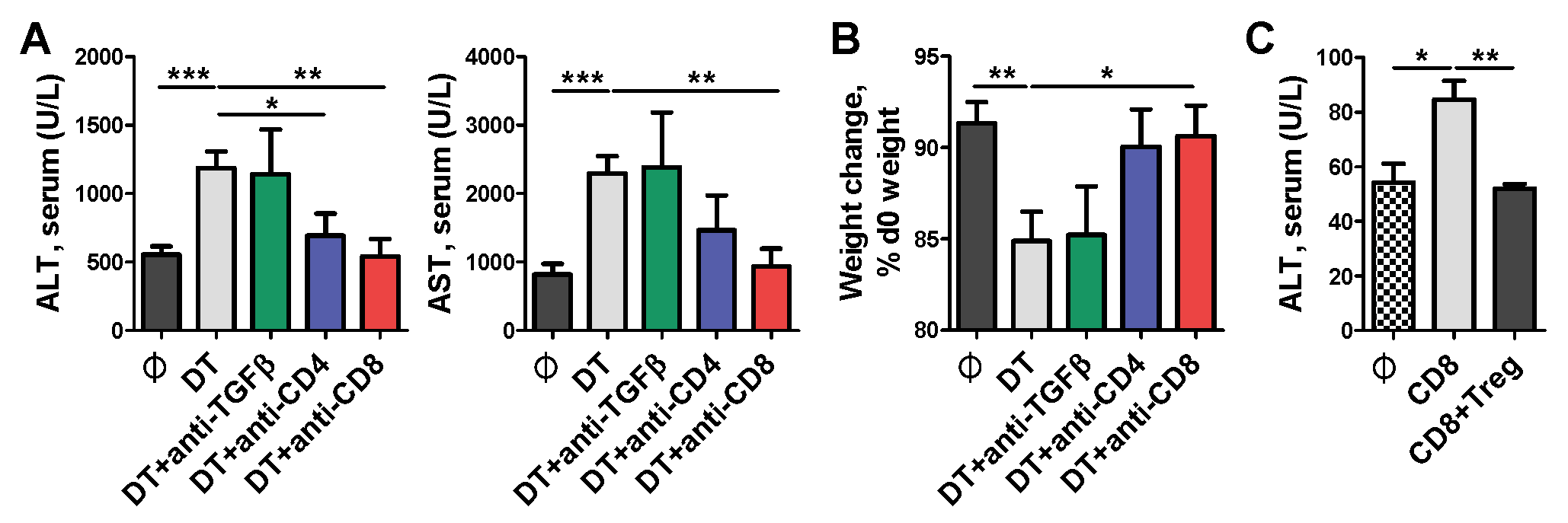

Supplement: S4 Fig — BALB/c DEREG mice were i.p. injected with either anti-TGFβ, anti-CD8 or anti-CD4 antibody 3 hours prior to infection. Mice were i.v. injected with 106 WT MCMV (pSM3fr-MCK-2fl clone 3.3) and treated i.p. with DT on day 0 and 1 or left untreated. Mice were analyzed on day 5 p.i. (A) AST and ALT levels were determined in the serum. Pooled data from 2 independent experiments are shown as mean ± SEM of n = 8–9 mice (B) Changes in the body weight on day 4 p.i. were determined as a percent of weight at the date of infection. Data are shown as mean ± SEM of n = 5–6 mice from one representative experiment. (C) BALB/c SCID mice were i.v. injected with 106 WT MCMV (pSM3fr-MCK-2fl clone 3.3) and at the same day of infection received 2x10^6^ CD8+ T cells from naive BALB/c mice alone or together with 1x10^6^ Treg cells. ALT levels were determined in the serum on day 5 p.i. Data are shown as mean ± SEM of n = 3–4 mice from one representative experiment. *p<0.05; **p<0.01; ***p<0.001 from two tailed, unpaired Student’s t-test. (TIF) [file ppat.1006345.s004.tif]

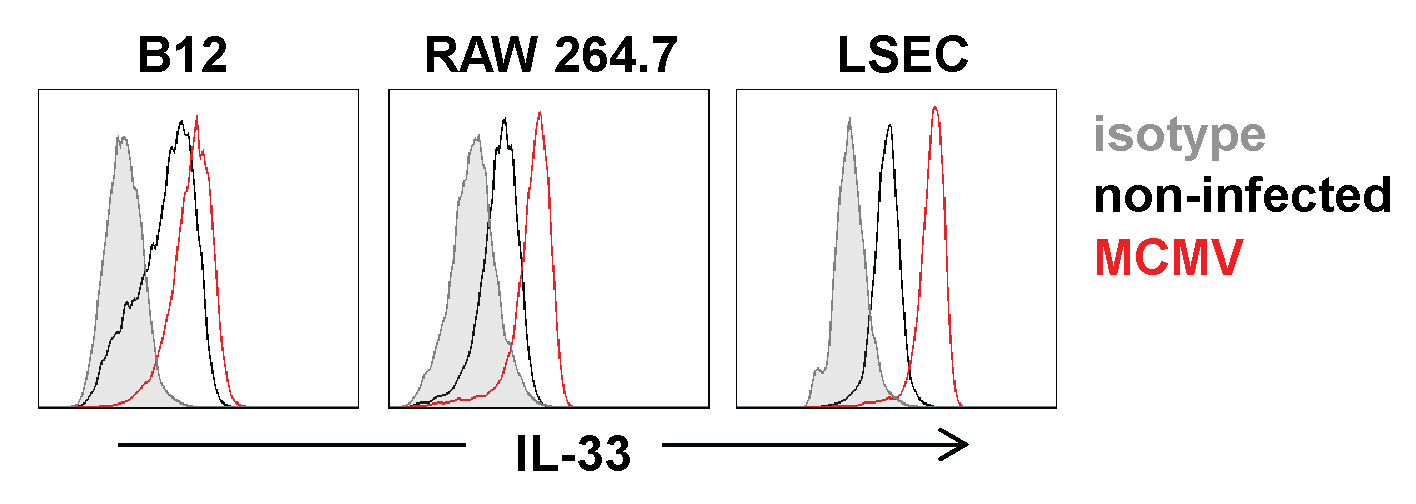

Supplement: S5 Fig — B12, RAW 264.7 and LSEC cells were infected with Δm138 MCMV or left uninfected. Cells were harvested 24 hours p.i. and stained for intracellular IL-33. Data are representative of three independent experiments. (TIF) [file ppat.1006345.s005.tif]

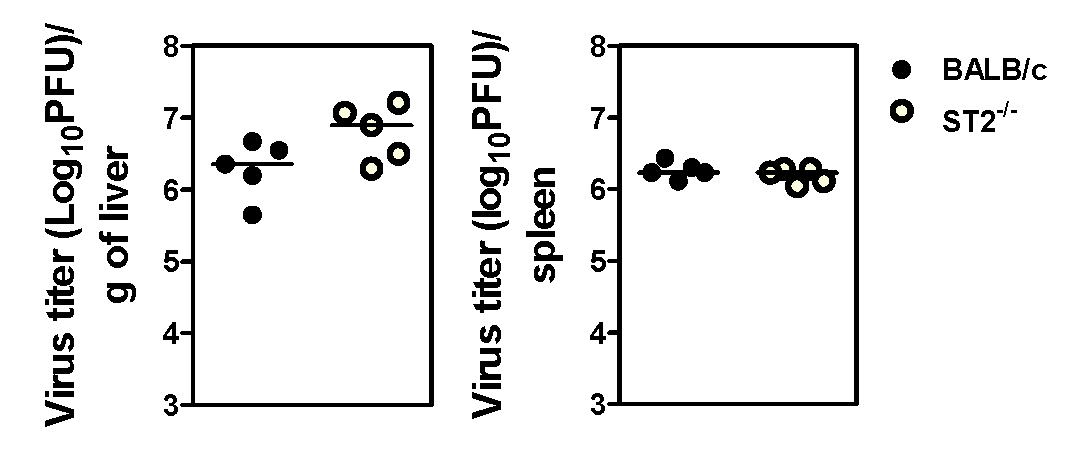

Supplement: S6 Fig — BALB/c and ST2-/- mice were i.p. injected with 5x10^4^ PFU of SGV MCMV. Viral titers in indicated organs 6 days post infection were determined by the plaque assay. A circle depicts the titer for each individual mouse; a small horizontal line indicates the median. n = 5 mice from one representative experiment out of two. (TIF) [file ppat.1006345.s006.tif]
